# ADAM8 silencing suppresses the migration and invasion of fibroblast-like synoviocytes via FSCN1/MAPK cascade in osteoarthritis

**DOI:** 10.1186/s13075-023-03238-w

**Published:** 2024-01-13

**Authors:** Kai Chen, Huaqiang Tao, Pengfei Zhu, Miao Chu, Xueyan Li, Yi Shi, Liyuan Zhang, Yaozeng Xu, Shujun Lv, Lixin Huang, Wei Huang, Dechun Geng

**Affiliations:** 1 Department of Orthopedics, The First Affiliated Hospital of Soochow University, Shizi Street 188, Suzhou, Jiangsu China; 2 Anesthesiology department, Suzhou Municipal Hospital (North District), Nanjing Medical University Affiliated Suzhou Hospital, Guangjj Road 242, Suzhou, Jiangsu China; 3 Department of Orthopedics, Hai’an People’s Hospital, Zhongba Road 17, Hai’an, Jiangsu China; 4 Department of Orthopedics, The First Affiliated Hospital of USTC, Division of Life Sciences and Medicine, University of Science and Technology of China, Lujiang Road 17, Hefei, An’hui China; 5 Department of Orthopedics, Yixing Peoples’s Hospital, Xincheng Road 1588, Yixing, Jiangsu China

**Keywords:** Osteoarthritis, Fibroblast-like synoviocytes, ADAM8, Inflammation, MAPK

## Abstract

**Objective:**

Osteoarthritis (OA) is a degenerative joint disease that affects elderly populations worldwide, causing pain and disability. Alteration of the fibroblast-like synoviocytes (FLSs) phenotype leads to an imbalance in the synovial inflammatory microenvironment, which accelerates the progression of OA. Despite this knowledge, the specific molecular mechanisms of the synovium that affect OA are still unclear.

**Methods:**

Both in vitro and in vivo experiments were undertaken to explore the role of ADAM8 playing in the synovial inflammatory of OA. A small interfering RNA (siRNA) was targeting ADAM8 to intervene. High-throughput sequencing was also used.

**Results:**

Our sequencing analysis revealed significant upregulation of the MAPK signaling cascade and ADAM8 gene expression in IL-1β-induced FLSs. The in vitro results demonstrated that ADAM8 blockade inhibited the invasion and migration of IL-1β-induced FLSs, while also suppressing the expression of related matrix metallomatrix proteinases (MMPs). Furthermore, our study revealed that inhibiting ADAM8 weakened the inflammatory protein secretion and MAPK signaling networks in FLSs. Mechanically, it revealed that inhibiting ADAM8 had a significant effect on the expression of migration-related signaling proteins, specifically FSCN1. When siADAM8 was combined with BDP-13176, a FSCN1 inhibitor, the migration and invasion of FLSs was further inhibited. These results suggest that FSCN1 is a crucial downstream factor of ADAM8 in regulating the biological phenotypes of FLSs. The in vivo experiments demonstrated that ADAM8 inhibition effectively reduced synoviocytes inflammation and alleviated the progression of OA in rats.

**Conclusions:**

ADAM8 could be a promising therapeutic target for treating OA by targeting synovial inflammation.

**Supplementary Information:**

The online version contains supplementary material available at 10.1186/s13075-023-03238-w.

## Introduction

Osteoarthritis (OA) is the most prevalent degenerative disease affecting both axial and peripheral joints in the human body [1–3]. It is marked by the gradual deterioration of articular cartilage, bone growth in the articular margin and subchondral area, and abnormal immune inflammation in the synovial microenvironment. Joint pain and swelling, along with dysfunction, are the primary symptoms experienced by patients [4–6]. Currently, the exact cause of OA remains unclear, and there are multiple clinical approaches to treating it. While oral NSAIDs are frequently utilized, their effectiveness cannot be improved through excessive use and may even lead to harmful side effects [7–9]. Thus, it is crucial to continue researching the pathogenesis and potential therapeutic targets of osteoarthritis.

In a joint, the two primary tissues are hyaline cartilage and synovium. Research has demonstrated that synovial inflammation is a significant pathological characteristic in the early stages of OA and is closely linked to the clinical symptoms experienced by patients [10, 11]. The primary manifestation of synovial lesions in OA is chronic and low-grade inflammation, and the microenvironment of synovial inflammation is involved in repairing tissue damage [12, 13]. Fibroblast-like synoviocytes (FLSs) are mesenchymal cells that reside in the synovium. Their abnormal activation and altered biological function are crucial in the formation and development of OA [14, 15]. Recent studies have shown that OA-FLSs share similar biological characteristics with FLSs in rheumatoid arthritis [16, 17]. Changes in their biological phenotype lead to abnormal proliferation and secretion of inflammatory factors in synovium, which accelerates the progression of OA.

In our experiment, we utilized IL-1β to disrupt FLSs and replicate the inflammatory microenvironment of OA. Through transcriptome sequencing, we discovered that the mitogen-activated protein kinase (MAPK) signaling pathway was notably upregulated in the IL-1β-interfered FLSs group relative to the control group. Previous studies have demonstrated that the MAPK signaling pathway, which serves as a vital cell information transmission pathway, plays a critical role in regulating a range of physiological and pathological effects, including cell growth and differentiation, and the inflammatory response. Numerous studies have demonstrated that MAPK signaling cascades play a crucial role in the proliferation and migration of FLSs, as well as the secretion of inflammatory factors [18–20]. Thus, we hypothesize that regulating the MAPK signaling pathway can effectively prevent the expression of FLS-specific biological phenotypes.

Furthermore, it was found that disintegrin and metalloproteinase domain-containing protein 8 (ADAM8) was significantly expressed in both IL-1β-induced FLSs and human synovial tissue. ADAM8 is a gene that codes for a protein. Research has demonstrated that this protein is involved in both calcium ion binding and metallopeptidase activity [21]. ADAM8 has been revealed to play a role in cancer cell invasion through its mediation of the MAPK pathway [22, 23]. Additionally, it has been reported that the expression of ADAM8 remains relatively stable under normal physiological conditions [24]. However, increased ADAM8 expression has been observed in response to inflammatory processes and in different types of cancers. Previously, Awan and colleagues found that ADAM8 plays a crucial role in promoting the proliferation, survival, and migration of hepatocellular carcinoma cells, ultimately leading to metastasis of the cancer [25]. In addition, Liu et al. reported that ADAM8 activates the NF-κB/MMP-13 signaling axis, leading to increased migration and invasion of chondrosarcoma cells [26]. These studies offer valuable insights into the regulation of OA-FLSs proliferation, invasion, and inflammatory factor secretion by ADAM8 in OA, which we can further explore. In our study, we blocked ADAM8 both in vivo and in vitro to observe how it affects the FLSs phenotype mediated by IL-1β and OA synovial inflammation in OA. Collectively, our aim was to offer fresh and valuable perspectives on the management of OA.

## Materials and methods

### Chemicals and reagents

Rat IL-1β protein (purity > 90%) was acquired from Sino Biological company (Beijing, China). RNA interference sequences for ADAM8 were purchased from GenePharma in Suzhou, China. Thermo Fisher Scientific (St. Louis, USA) provided DMEM/F12 for cell culture, as well as fetal bovine serum (FBS) and phosphate buffer saline (PBS). Monosodium iodoacetate (MIA) was obtained from Sigma in Gillingham, UK. Antibodies included anti-MMP-13, TNF-α, IL-6, COX2, P-JNK, JNK, P-ERK, ERK, P-P38, P38, and β-actin antibodies were obtained from Abcam, UK. The antibodies included anti-ADAM8 and MMP-1 were obtained from ThermoFisher, USA. BDP-13176, a FSCN1 inhibitor, was purchased from MedChemExpress (New Jersey, USA).

### Cell culture

Rat primary synoviocytes were obtained from Procell Life Science & Technology Company (Procell, Wuhan, China) and cultured in DMEM/F12 medium supplemented with 10% fetal bovine serum. The cells were cultured at 37℃ with 5% CO_2_ and cells from passages 4–6 were selected for use in experiments.

### Cell transfection

The transfection process was carried out using GP-transfect-Mate from GenePharma, China, following the manufacturer’s protocol. Initially, FLSs were seeded in a plate at the appropriate density and allowed to adhere overnight. The cells were then transfected with 20 nM siRNA, and the medium was changed to complete DMEM/F12 culture medium overnight, which was maintained for 72 h. Total samples were harvested, and the knockdown efficiency of ADAM8 was detected using immunofluorescence staining and RT-PCR. The siRNA sequences used in this study can be found in Table S1.

### RNA sequencing

In our studies, we utilized IL-1β as an intervention for FLSs over a period of 6 h. Subsequently, we extracted total RNA by a TRIzol reagent (Beyotime, China). The extracted samples were then sent to OE Biotech in Suzhou, China, for further sequencing. The FPKM of each gene was calculated using Cufflinks, and the read count of the detected gene was obtained. Differential expression genes were analyzed using the R package. And the significance threshold was *P* < 0.05 and the fold change threshold was > 1.5 or < 0.5. To detect ADAM8 downstream, we utilized the same sequencing operation following successful transfection of siADAM8 with high efficiency.

### Quantitative real-time PCR assay

In this study, we isolated total RNA from FLSs subjected to different interventions using TRIzol reagent (Beyotime, China) and quantified the RNA density with a NanoDrop 2000 system (Thermo Fisher Scientific). Then, we extracted complementary DNA (cDNA) from the isolated RNA through reverse transcription. To amplify the reaction system, we combined 10 μl of qPCR Master Mix, 0.5 μl of forward and reverse primers, 2 μl of cDNA, and 7 μl of nuclease-free ddH_2_O. We used the comparative 2^−ΔΔCq^ method to determine the folding changes in mRNA expression. The gene primer sequences used in this study are listed in Table S2.

### Western blotting

After collecting FLSs with different interventions, the cells were rinsed twice with PBS and lysed using RIPA buffer (Beyotime, China). The resulting lysate supernatant lysate was collected, and the protein concentration was determined by the use of a BCA kit (Beyotime, China). Next, all proteins from the various experimental groups were transferred onto a polyvinylidene chloride (PVDF) membrane. After the barrier buffer was sealed in, the membrane was incubated with primary antibody solutions for 12 h. Following this, the membrane was rinsed for 20 min and the corresponding secondary antibody solution was added for another 1 h. Finally, protein band images were visualized and the protein gray was quantified by Image Lab software version 3.0 (Bio-19 Rad, USA).

### Wound-healing assay

In this study, we prepared single-cell suspensions of FLSs under various treatment conditions and inoculated them into 6-well plates at a density of 1 × 10^6^ cells per well. We then used a P-200 pipette tip to create a scratch on the surface of the plates, allowing the FLSs to adhere to the walls of the orifice plate. Any shed FLSs cells were washed away by PBS, and the remaining cells were then incubated in serum-free DMEM/F12. We observed the cells at scratch site under an inverted microscope (Carl Zeiss, Germany) after 0 and 24 h.

### Migration and invasion assay

After treating FLSs with IL-1β and siADAM8, we implanted them into the upper chamber of a transwell at a concentration of 2 × 10^4^ cells per well, using 100 μl of serum-free DMEM/F12. Simultaneously, we added 600 μl of DMEM/F12 with 20% FBS to the lower lumen of a 24-well plate (BD Bioscience, USA). In contrast to migration analysis, detecting the invasion of FLSs involves an extra layer of matrix glue in the upper chamber of the transwell. Following a 24-h period, the FLSs were fixed with 4% paraformaldehyde for 20 min and rinsed. Subsequently, the upper cells were carefully wiped with a damp cotton swab, while the FLSs in the lower chamber were stained with crystal violet solution for half an hour. Finally, the FLSs in the lower chamber were rinsed with PBS for 5 min. Pictures were taken under an inverted microscope (Carl Zeiss, Germany) and the number of migrating and invading FLSs was evaluated and calculated with ImageJ software (Media Cybernetics, USA).

### Immunofluorescence assay

After treating FLSs with various interventions, the cells were fixed using 4% paraformaldehyde and permeabilized with 0.2% Triton X-100 (Beyotime, China) on ice. Then, cells were blocked with QuickBlock buffer (Beyotime, China) for another hour. The cells were incubated primary antibodies against ADAM8, IL-6, and TNF-α protected from light at 4 °C overnight, followed by incubation with F-actin (Yeasen, China). To stain cell nuclei, DAPI (Beyotime, China) was used for a duration of 10 min. The FLSs were then observed and photographed using a Zeiss laser scanning microscope (LSM 510, Zeiss).

### Management of the synovial tissue in OA patients

All human experiments conducted in this study were in compliance with the ethical standards set by the Hai’an People's Hospital. Synovial tissues were obtained from two groups of patients: those who underwent total knee arthroplasty due to knee OA (OA group, *n* = 5, 4 females, 1 male; median age, 73 years; age range, 62–81 years) and those who got arthroscopic knee surgery because of anterior cruciate ligament injury (ACL group, *n* = 5, 2 females, 3 males; median age, 42 years; age range, 31–49 years). The synovial tissue was excised and sectioned into uniform pieces. which were then washed three times with a PBS solution before being embedded and cut into blocks for subsequent staining.

### Animals and OA model induction

This study was conducted in accordance with the ethical guidelines set forth by the Soochow University Ethics Committee, which approved all animal experiments and procedures. Eighteen 8-week-old male Sprague Dawley rats were chosen as subjects for this study. They were all fed and maintained under standard conditions, which included maintaining a temperature range of 22–25℃, ensuring proper ventilation, and providing a 12-h light and dark cycle. In our study, animals were divided into three groups through random assignment: the sham surgery group, the MIA group, and the ADAM8 intervention group (MIA + siADAM8, joint cavity injection). The OA model group undertook a single intraarticular injection of MIA (Sigma, USA) 50 μl, while the sham operation group got an equivalent amount of saline. In the drug intervention group, rats were injected with 50 μl of siADAM8 at a concentration of 300 μM into their knee joint on the 7th, 14th, and 21st day of modeling. The modeling process lasted for 28 days until the rats were euthanized.

### Immunohistochemical staining

We obtained knee tissues from three groups of rats and subjected them to decalcification for a period of 30 days, utilizing 10% ethylenediaminetetraacetic acid (EDTA, Sigma, USA). Subsequently, we performed gradient dehydration and embedding. The resulting embedded tissue wax blocks were sliced into 7-μm sections and stained with hematoxylin and eosin (H&E), as well as safranin O, in accordance with established protocols. Subsequently, we utilized immunohistochemical (IHC) staining to detect the presence of ADAM8, IL-1β, TNF-α, IL-6, MMP-1, and MMP-13. These primary antibodies were chosen for incubation and staining purposes. To quantify the number of positive cells, we utilized ImageJ software (Media Cybernetics, USA) for analysis.

### Statistical analysis

The data values were expressed as mean ± standard deviation. To determine whether there was a significant difference between the two groups, a *t*-test was utilized for analysis. Additionally, multiple comparisons were determined through the use of a Tukey test following a one-way analysis of variance. GraphPad Prism8 was used for all statistical analysis. A *p*-value of less than 0.05 was considered statistically significant, while a *p*-value of less than 0.01 was deemed highly significant.

## Results

### The MAPK signaling cascade is involved in the processes of FLSs treated with IL-1β

**Fig. 1:**
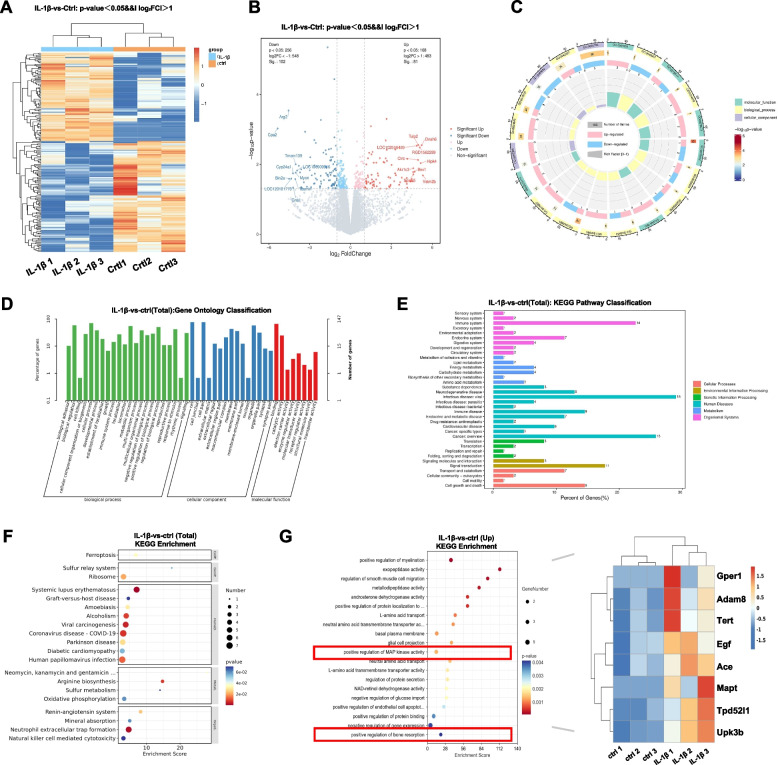
The MAPK signaling cascade is involved in the processes in FLSs treated with IL-1β. **A** Differentially expressed mRNAs in FLSs in response to IL-1β are illustrated as a heat map. **B** Volcano map showing differentially expressed mRNAs. **C-D** Gene ontology classification. **E** KEGG pathway classification. **F** Bubble map of KEGG enrichment analysis (Total). **G** Bubble map of KEGG enrichment analysis (Up) and correlational heatmap of different samples and MAP kinase activity and bone resorption-related markers in different samples

To model the synovial inflammatory microenvironment of OA, we utilized IL-1β to intervene FLSs. We report on our sequencing results which indicate the presence of 183 differentially expressed genes (DEGs) in IL-1β-interfered FLSs. Specifically, we found that 81 of these genes were upregulated, while 102 were downregulated (Fig. 1A). The volcano diagram illustrates the distribution of unaffected and DEGs in FLSs, both with and without IL-1β intervention. Additionally, the diagram includes the names of the top 10 upregulated and downregulated DEGs (Fig. 1B). The expressions of these DEGs were detected using PCR, and the results obtained were consistent with those demonstrated by sequencing (Figure S1). The gene ontology classification analysis showed that there were differences in the enrichment of FLSs biological processes, intracellular components, and molecular functions following IL-1β intervention (Fig. 1C-D). As shown, we observed a significant impact on the biological adhesion function and binding ability of the FLSs. Though KEGG pathway classification, we observed that IL-1β-induced FLSs intervention was associated with the development of infectious diseases, the immune system, and cell growth and death (Fig. 1E-F). Upon analyzing the up-regulated signaling pathways, we observed a significant increase in MAP kinase activity. This increase is attributed to the differential genes involved, such as Gper1, ADAM8, and Tert, among others. This observation suggests that the MAPK signaling cascade MAY play a crucial role in IL-1β-induced inflammation models in vitro (Fig. 1G).

### ADAM8 is upregulated in FLSs after treatment with IL-1β.

**Fig. 2:**
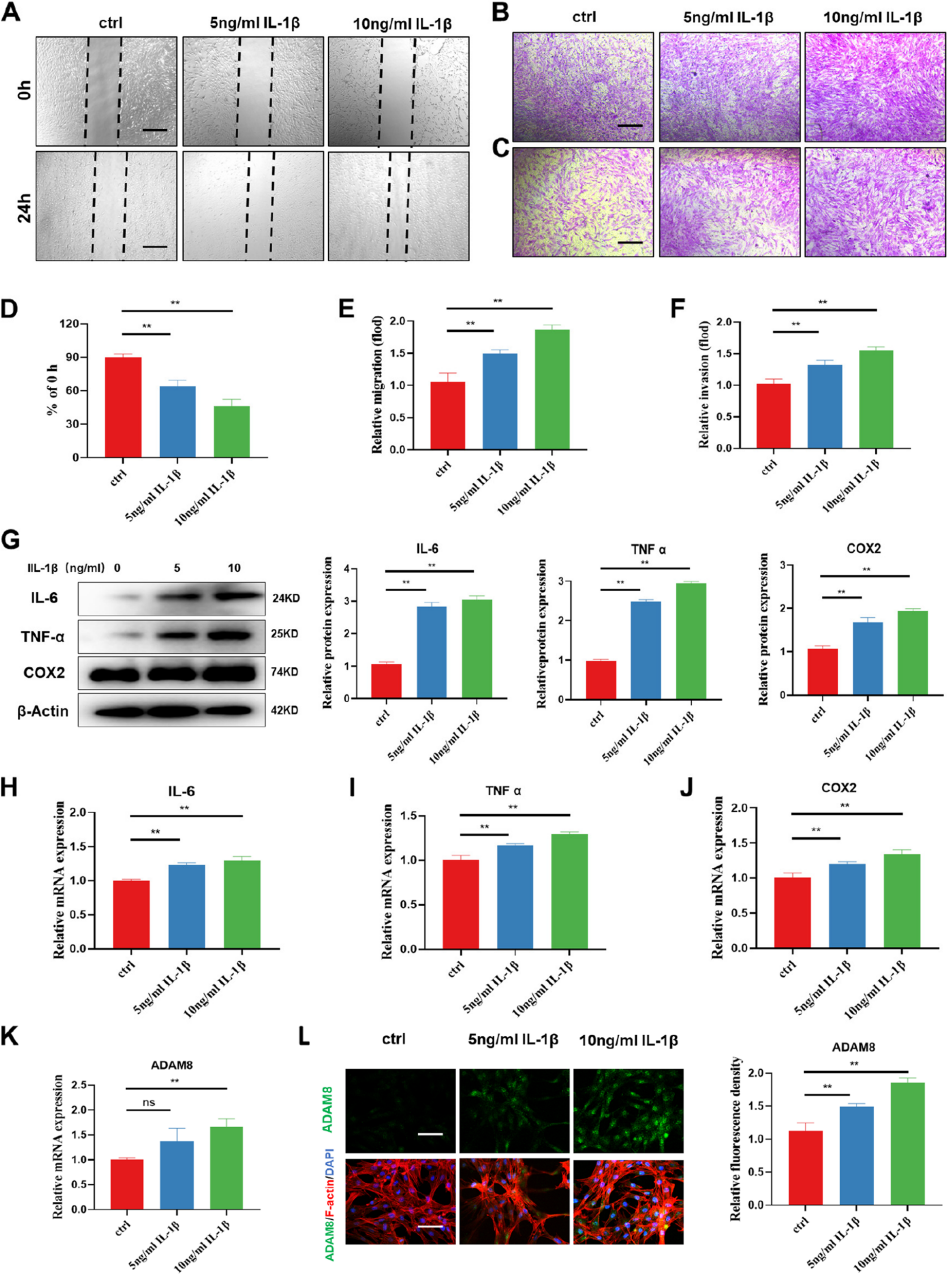
ADAM8 is up-regulated in FLSs after treatment with IL-1β. **A**, **D** Wound healing assays and quantitative analysis of the migration abilities of FLSs with different concentrations of IL-1β. **B**, **E** Transwell assays and quantitative analysis of the migration ability of FLSs with different concentrations of IL-1β. **C**, **F** Transwell assays and quantitative analysis of the invasion abilities of FLSs with different concentrations of IL-1β. **G** Western blot and quantitative analysis of IL-6, TNF-α, and COX-2 in FLSs after treatment with IL-1β. **H-J** qRT-PCR analysis of the mRNA expression levels of IL-6, TNF-α, and COX2. **K** qRT-PCR analysis of the mRNA expression levels of ADAM8. **L** Immunofluorescence staining of ADAM8 in FLSs. Scale bar = 200 µm. *P < 0.05; **P < 0.01

In our study, we conducted an experiment using IL-1β with varying concentrations (0, 5, and 10 ng/ml) to intervene with FLSs. To evaluate cell migration activity in vitro, we utilized a scratch assay, which proved to be an effective simulation. Our findings indicate that IL-1β promotes FLSs migration in a concentration-dependent manner (Fig. 2A and D). Upon conducting additional in vitro crystal violet staining, it was observed that IL-1β enhances the invasion and migration of FLSs in a concentration-dependent manner (Fig. 2B-C and E-F). Matrix metalloproteinases (MMPs) are enzymes that break down collagen and have various functions, including promoting cell proliferation, migration, and differentiation. Furthermore, western blot results found that the expressions of inflammation-related markers, including IL-6, TNF-α, and COX2 were significantly higher in experimental FLSs compared to the control group (Fig. 2G), which was consistent with our PCR results (Fig. 2H-J). To further investigate the impact of the MAPK signaling cascade on FLS-specific biological phenotypes, we utilized adezmapimod, a potent inhibitor of MAPK, to intervene in IL-1β-induced FLSs. Based on the CCK8 results, we employed a concentration of 50 μM of adezmapimod for this intervention. Western blot analysis revealed that adezmapimod effectively inhibited the expression of P-ERK and P-P38 in FLSs. Moreover, we observed that the inhibition of MAPK significantly suppressed the invasion, migration, and inflammatory expression of FLSs stimulated by IL-1β (Figure S2), which further illustrated the role of the MAPK cascade in IL-1β stimulated FLSs. The transcriptome differential gene analysis revealed that IL-1β increased the expression of ADAM8. Additionally, we conducted RT-PCR and observed a gradual upregulation of ADAM8 following the intervention of IL-1β (Fig. 2K). Immunofluorescence staining confirmed the upregulation of ADAM8 in FLSs that had been stimulated by IL-1β (Fig. 2L).

**Fig. 3:**
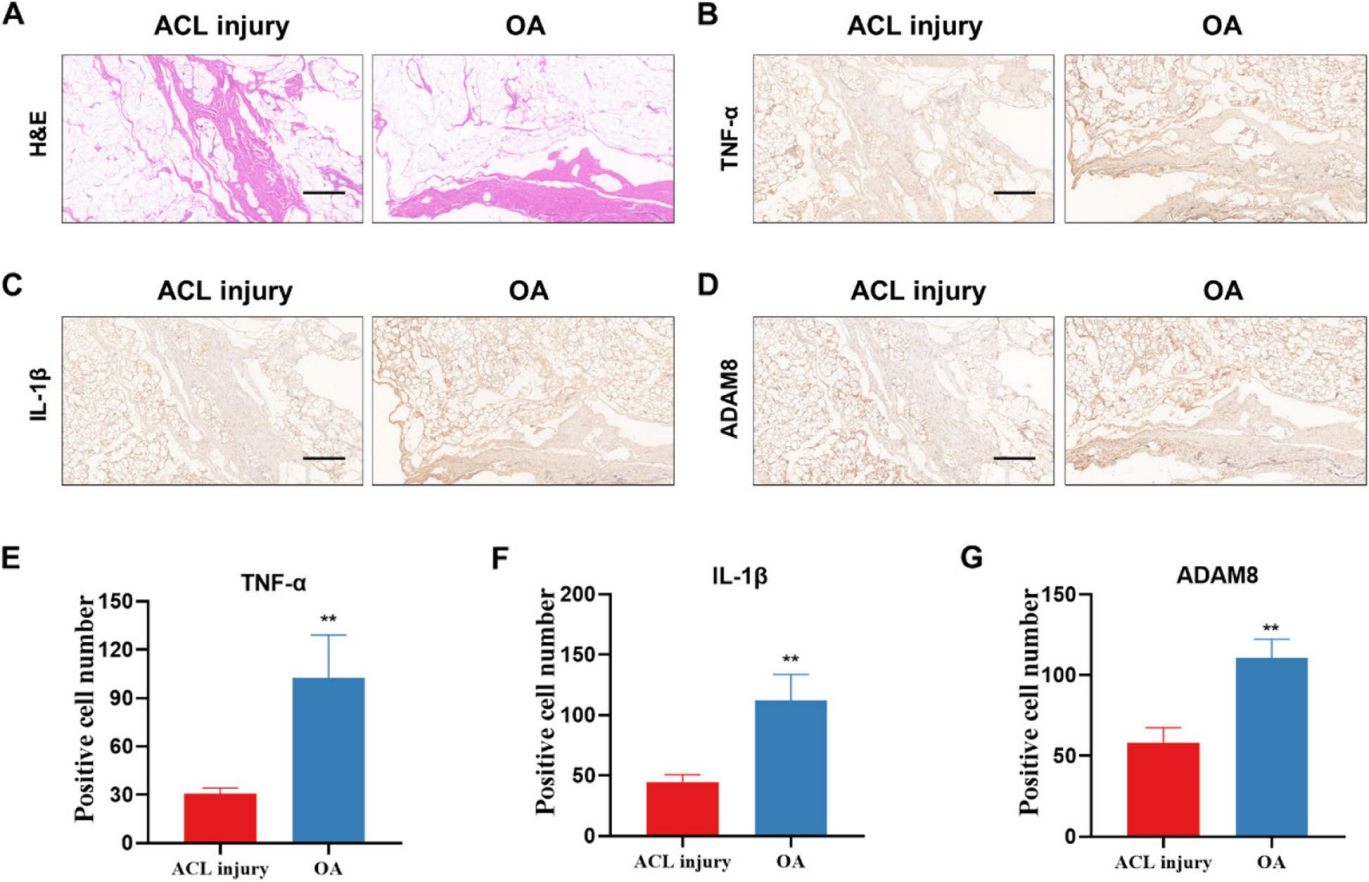
The expression of ADAM8 was found to be increased in the synovium of patients with OA. **A** H&E staining of synovial tissues in ACL injury and OA patients. **B-D** TNF-α, IL-1β, and ADAM8 IHC staining of synovial tissues in ACL injury and OA patients. **E–G** Quantification of IHC staining for TNF-α, IL-1β, and ADAM8. Scale bars, 100 µm. **P < 0.01

ADAM8 expression was also detected in synovial tissues of patients with OA. Studies have revealed variations in the biological characteristics of synovial tissue between patients with ACL injuries and patients with OA [27, 28]. In our study, we utilized ACL patients as a model to simulate the condition of individuals without OA as reported in previous studies [29]. Following the acquisition of informed consent from the patients, we surgically procured synovial tissue from ACL patients. This enabled us to distinguish it from postoperative synovium obtained from patients with severe OA. The aim was to observe the genes that exhibit significant changes during the progression of OA. We found that the expressions of inflammatory cytokines TNF-α and IL-1β were significantly increased in the synovium of OA patients compared with ACL (non-OA) patients, as well as the expression of ADAM8, suggesting that ADAM8 may be involved in the synovium progression of OA (Fig. 3).

### ADAM8 blockade suppresses FLS migration and invasion by inhibiting MMPs

**Fig. 4:**
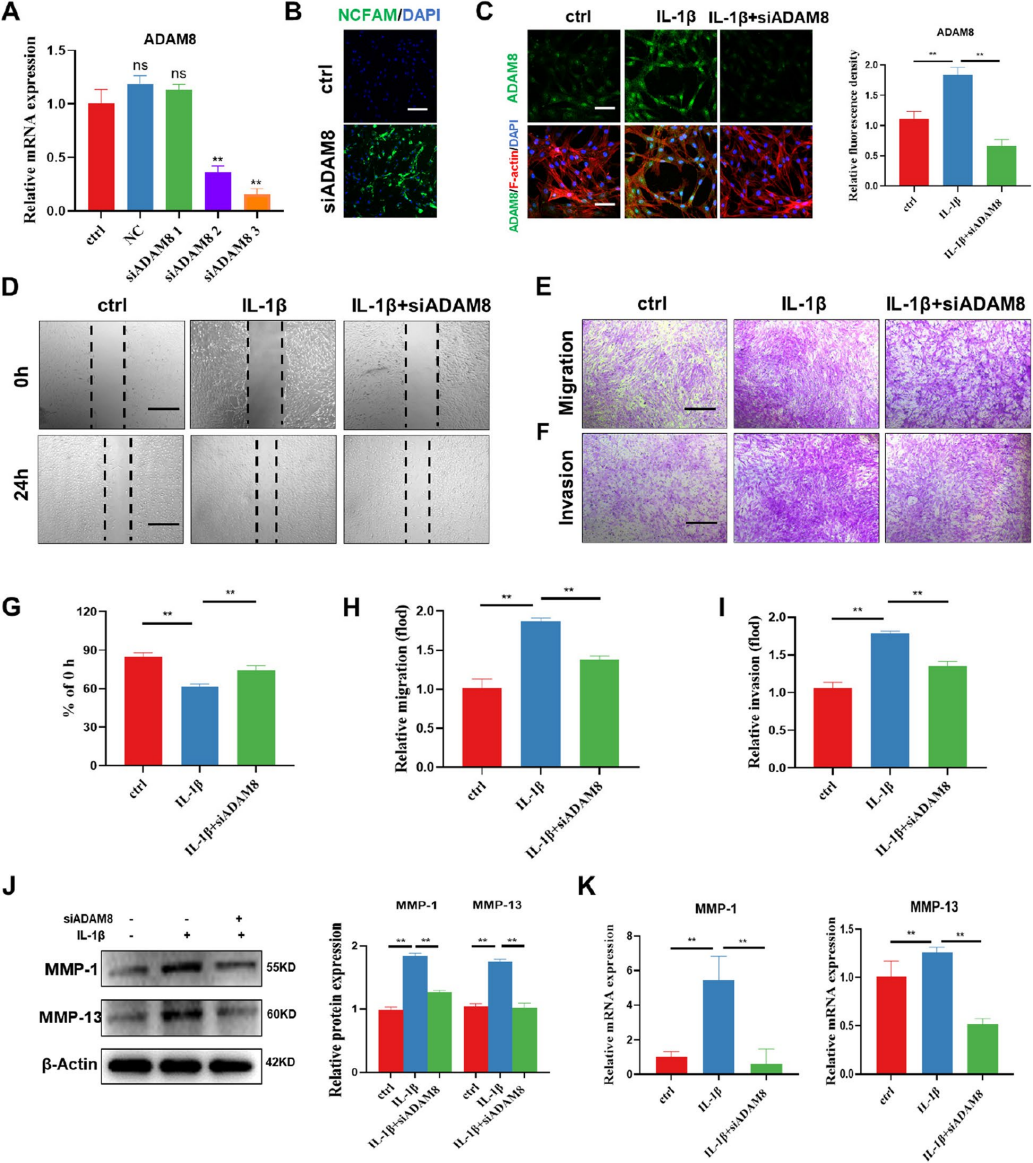
ADAM8 blockade suppresses FLS migration and invasion by inhibiting MMPs. **A** qRT-PCR analysis of the mRNA expression levels of ADAM8. **B** ADAM8 transfection efficiency was detected by fluorescence assay. **C** Immunofluorescence staining of ADAM8. **D**, **G** Wound healing assays and quantitative analysis of the migration ability of FLSs. **E**, **H** Transwell assays and quantitative analysis of the migration ability of FLSs. **F**, **I** Transwell assays and quantitative analysis of the invasion ability of FLSs with different concentrations of IL-1β. **J** Western blot and quantitative analysis of MMP-1 and MMP-13. **K** qRT-PCR analysis of the mRNA expression levels of MMP-1 and MMP-13. Scale bar = 200 µm. *P < 0.05; **P < 0.01

To further investigate whether ADAM8 regulates the biological behavior of FLSs, we utilized siRNA to manipulate the expression level of ADAM8 in FLSs (Fig. 4A). The RT-PCR results indicated that siADAM8 3 had the most significant impact on ADAM8 knockdown in FLSs, which was consistent with the immunofluorescence findings (Fig. 4B-C). Furthermore, our results indicate that ADAM8 inhibition led to a significant decrease in the migration of FLSs, as demonstrated by scratch assays and migration analysis (Fig. 4D-E and G-H). Additionally, our FLS invasion assays revealed that siADAM8 treatment attenuated the invasive properties of FLSs under IL-1β intervention (Fig. 4F and I). Previous research has demonstrated a correlation between the invasion and migration ability of cells and the expression and secretion of MMPs. In this study, we further investigated the impact of siADAM8 intervention on the expression of MMPs in FLSs. Our western blot results indicated ADAM8 silencing inhibited the expression of MMP-1 and MMP-13, which was consistent with the RT-PCR results (Fig. 4J-K). Our findings indicate that ADAM8 has the potential to impede the invasion and migration of FLSs by regulating the levels of MMPs.

### ADAM8 blockade suppresses FLS-mediated inflammation by inhibiting MAPK signaling cascades

**Fig. 5:**
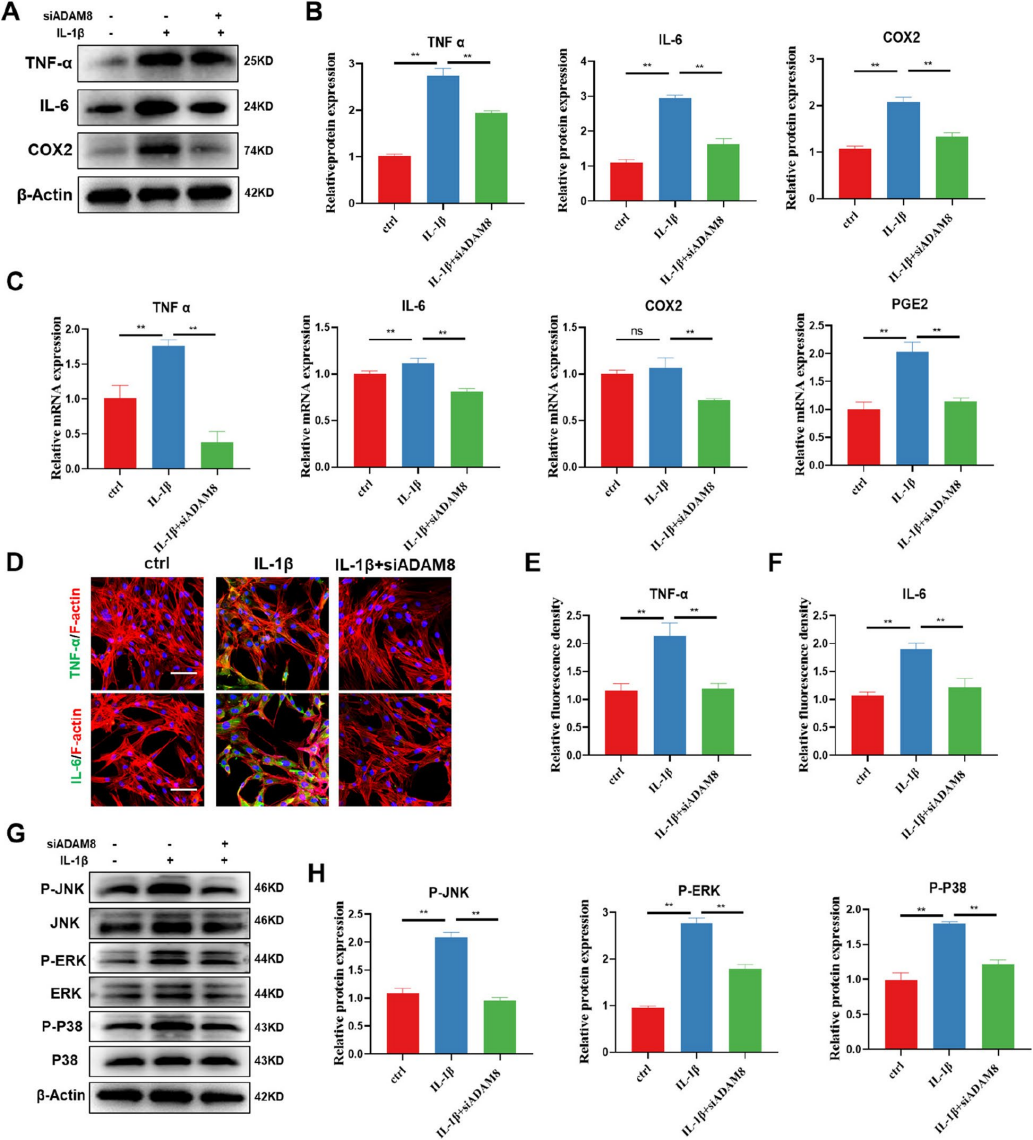
ADAM8 blockade suppresses FLS inflammation by inhibiting MAPK signaling cascades. **A** Western blot and quantitative analysis of TNF-α, IL-6 and COX2 after treatment with siADAM8. **H** qRT-PCR analysis of the mRNA expression levels of TNF-α, IL-6, COX2, and PGE2. **D-F** Immunofluorescence staining and quantitative analysis of TNF-α and IL-6 after treatment with siADAM8. **G** Western blot of P-JNK, JNK, P-ERK, ERK, P-P38, and P38 after treatment with siADAM8. **H** Quantitative analysis of P-JNK/JNK, P-ERK/ERK, and P-P38/P38. *P < 0.05; **P < 0.01

The development of OA is heavily influenced by inflammatory disorders within the synovial microenvironment [30]. To expand on this, we conducted an investigation into the impact of ADAM8 intervention on the regulation of FLSs inflammation. In our study, we discovered that inhibiting ADAM8 was effective in reducing the expression of various inflammatory cytokines, including TNF-α, IL-6, and COX2 under IL-1β intervention (Fig. 5A-B). Furthermore, our RT-PCR analysis revealed that ADAM8 blockade inhibit gene expression of TNF-α, IL-6, COX2, and PGE2 (Fig. 5C). Our immunofluorescence results also showed that inhibition of ADAM8 suppressed the expression of TNF-α and IL-6 (Fig. 5D-F). The GSEA analysis revealed that the MAPK signaling pathway played a role in the inflammatory response of FLSs induced by IL-1β (Figure S3). We then investigated the impact of ADAM8 inhibition on the MAPK pathway. The western blot results, as illustrated in Fig. 5G-H, indicate that the expression levels of P-JNK, P-ERK, and P-P38 were considerably upregulated following IL-1β intervention. However, after ADAM8 knockdown treatment by FLSs, the expressions of these phosphorylated signaling proteins were significantly inhibited. This finding suggests that ADAM8 may act as an inhibitor of FLS-mediated inflammation through the MAPK signaling pathway.

### ADAM8 blockade prevents IL-1β-stimulated biological phenotypes specific to FLSs by targeting FSCN1

**Fig. 6:**
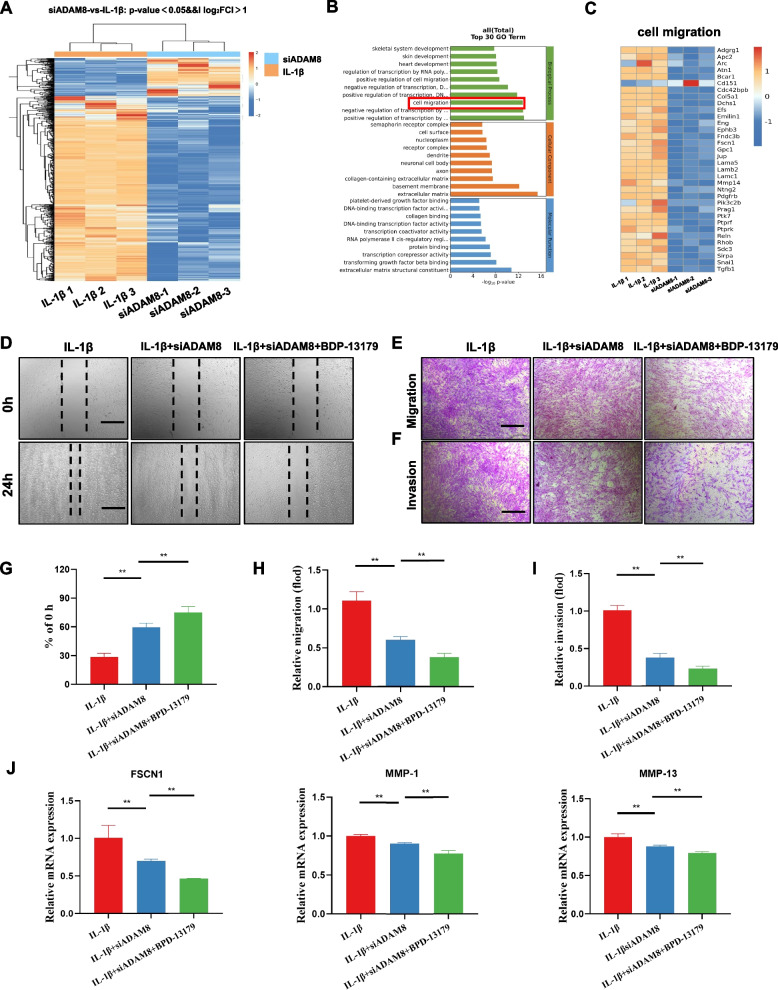
ADAM8 blockade prevent IL-1β-stimulated biological phenotypes specific of FLSs by targeting FSCN1. **A** The differentially expressed mRNAs in FLSs between IL-1β group and IL-1β + siADAM8 are illustrated as a heat map. **B** Gene ontology classification. **C** The heat map shows the detected differential genes associated with cell migration. **D**, **G** Wound healing and quantitative analysis of the migration ability of FLSs. **E**, **H** Transwell assays and quantitative analysis of the migration ability of FLSs. **F**, **I** Transwell assays and quantitative analysis of the invasion ability of FLSs with different concentrations of IL-1β.** J** qRT-PCR analysis of the mRNA expression levels of FSCN1, MMP-1, and MMP-13. Scale bar = 200 µm. *P < 0.05; **P < 0.01

To investigate the molecular mechanism of ADAM8 in regulating the biological behavior of FLSs, RNA sequencing was conducted. In this study, we designated the IL-1β intervention FLSs as the control group and the IL-1β + siADAM8 group as the experimental group for treatment comparison. Our study presents sequencing results that demonstrate the existence of 762 DEGs in FLSs subjected to the siADAM8 intervention compared with FLSs only stimulated by IL-1β. Further analysis revealed that 130 of these genes were upregulated, while 632 were downregulated (Fig. 6A). GO enrichment analysis showed that the DEGs were mainly classified in the cell migration signal category, where we observed 34 enriched DEGs (Fig. 6B). Among these differentially expressed genes, FSCN1 showed significant downregulation, which has previously been shown to be involved in regulation of the FLS biological phenotype (Fig. 6C). Furthermore, we used BDP-13176, a potent inhibitor of FSCN1, in combination with siADAM8 to intervene in the IL-1β-induced FLSs. Our results indicated that siADAM8 significantly inhibited FLS migration and invasion. Interestingly, the inhibitory effect was further enhanced when combined with BDP-13176 (Fig. 6D-I). These findings suggest that FSCN1 may play a crucial role downstream of ADAM8 in regulating the biological behavior of FLSs. Our RT‒PCR results indicate that siADAM8 inhibited FSCN1, MMP1, and MMP13 expression. Moreover, when combined with BDP-13176, the gene expression levels of FSCN1, MMP1, and MMP13 were further downregulated (Fig. 6J). Furthermore, we investigated the impact of intervening in FSCN1 on the MAPK signaling cascade. Our western blot analysis demonstrated that inhibition of FSCN1 in IL-1β-stimulated FLSs led to a significant reduction in the expression of P-ERK and P-P38 (Figure S4). These findings suggested that MAPK cascade may play a crucial role in ADAM8/FSCN1-mediated regulation of FLSs.

### Inhibition of ADAM8 alleviates OA progression and synovial inflammation in vivo

**Fig. 7:**
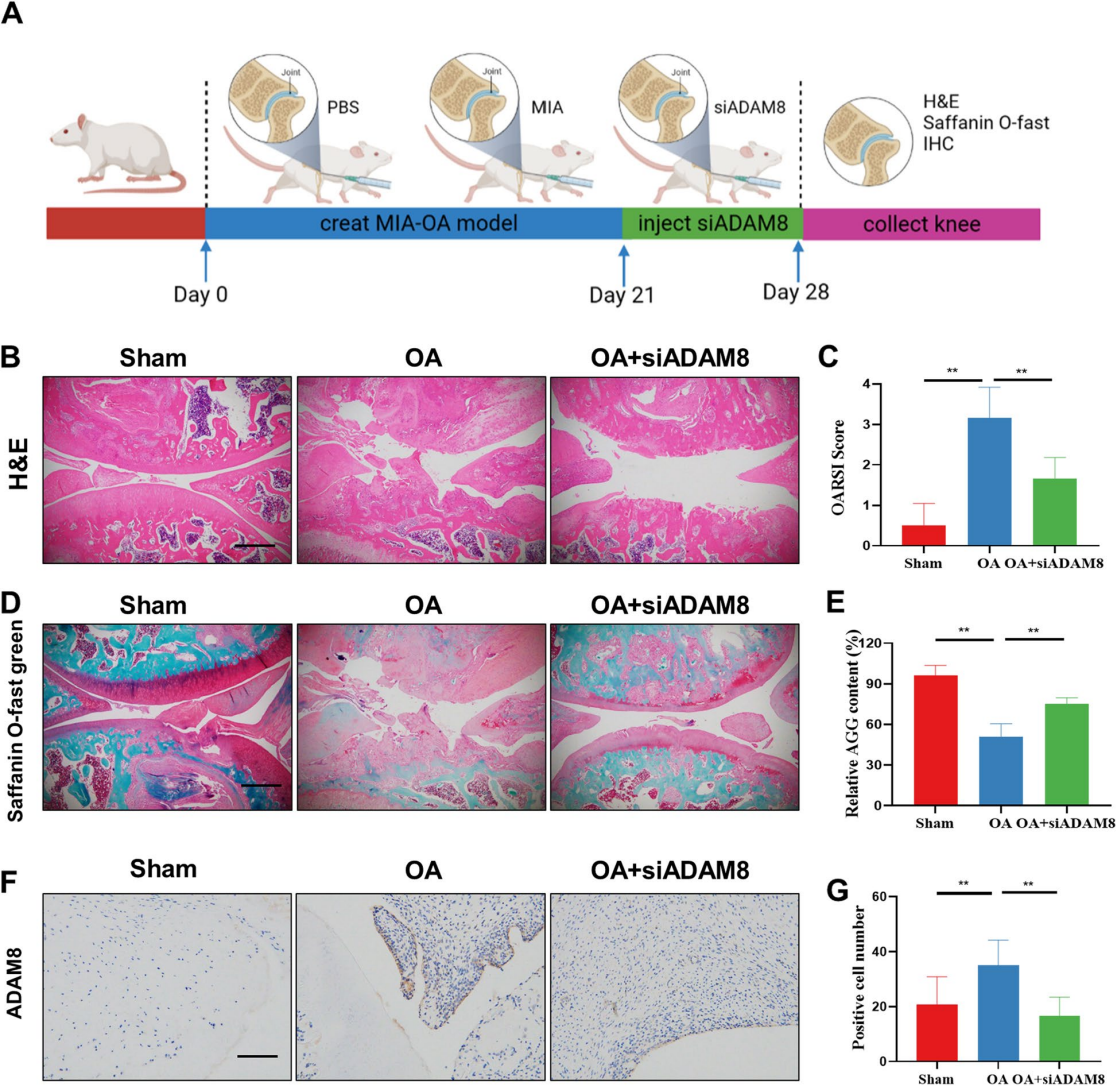
Inhibition of ADAM8 alleviates OA progression in vivo. **A** Schematic diagram of the in vivo experiments. **B**-**C** H&E staining and OARSI score of rat knee joint. **D**-**E** Safranin O staining of rat knee joint. **F**-**G** Immunohistochemical staining and quantitative analysis for ADAM8 of decalcified bone sections. Scale bars, 200 µm. *P < 0.05; **P < 0.01

As previously reported, we created a rat model of OA by injecting MIA into the joint. In addition, we administered an interventional therapy by injecting siADAM8. The experimental procedure is depicted in Fig. 7A. In order to assess inflammation and bone erosion, H&E staining was performed on the knee joints of rats in every group (Fig. 7B-C). Additionally, to evaluate cartilage damage, safranin O staining was also conducted (Fig. 7D). After conducting our study, we discovered that the knee cartilage experienced severe erosion as a result of MIA modeling. However, we found that ADAM8 treatment was able to effectively reverse this phenomenon and delayed the progression of OA (Fig. 7C and E). The results of ADAM8 immunohistochemical staining showed that injecting siRNA into the articular cavity effectively reduced the expression of ADAM8 in the synovium (Fig. 7F-G).

**Fig. 8:**
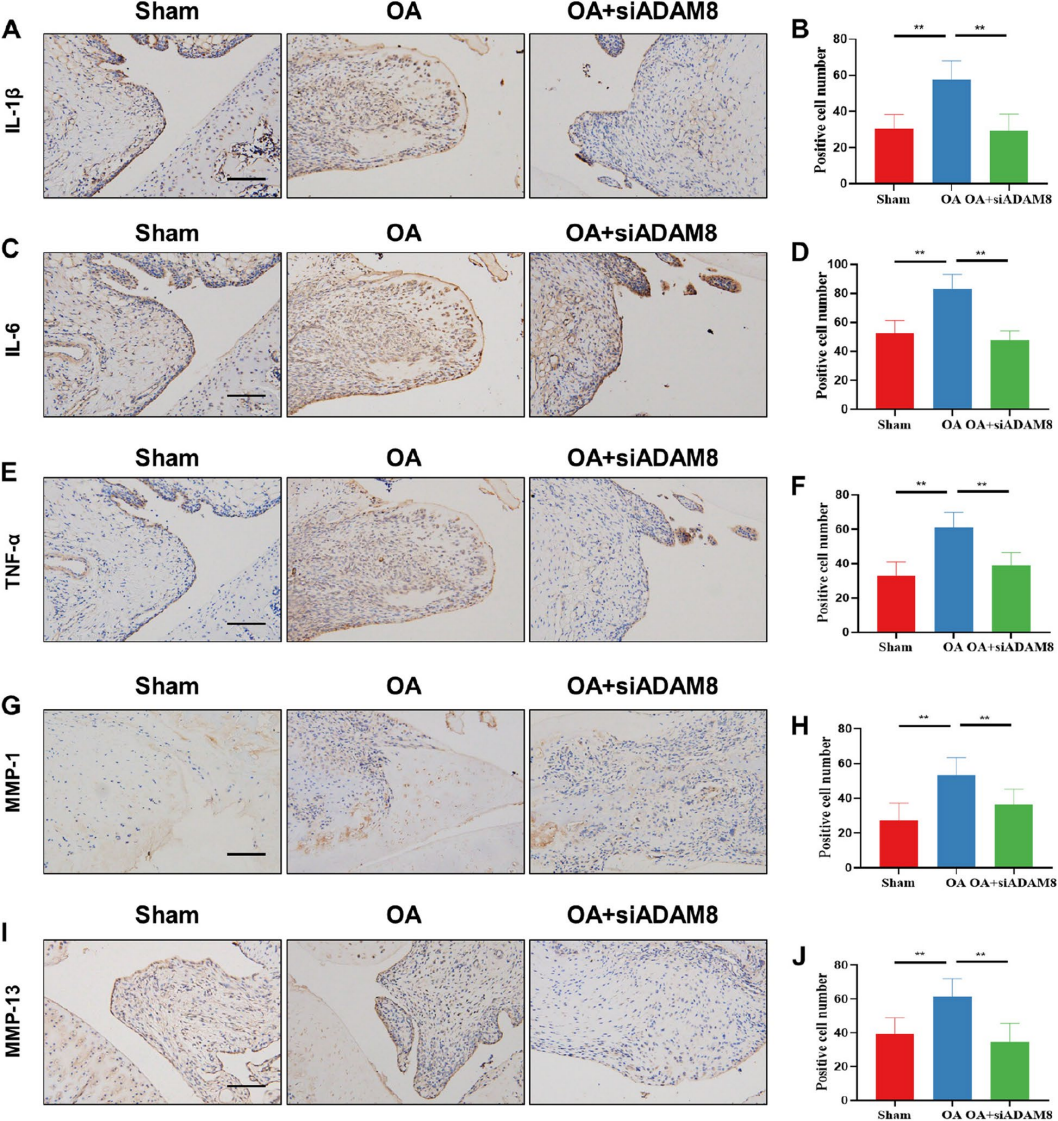
Inhibition of ADAM8 alleviates synovitis and MMPs in vivo. **A**, **B** IL-1β IHC staining and quantitative analysis of synovial tissues in OA rats. **C**, **D** IL-6 IHC staining and quantitative analysis of synovial tissues in OA rats. **E**, **F** TNF-α IHC staining and quantitative analysis of synovial tissues in OA rats. **G**, **H** MMP-1 IHC staining and quantitative analysis of synovial tissues in OA rats. **I**, **J** MMP-13 IHC staining and quantitative analysis of synovial tissues in OA rats. Scale bars, 200 µm. *P < 0.05; **P < 0.01

Our findings align with the in vitro results, as we observed a significant decrease in the expression of the inflammation-related factors TNF-α, IL-1β, and IL-6 in the synovium of OA rats following ADAM8 inhibition (Fig. 8A-F). siADAM8 was injected into the articular cavity of MIA-treated rats, and subsequent immunohistochemical staining revealed a significant decrease in MMP-1 and MMP-13 expressions in the rats’ synovium (Fig. 8G-J). These findings align with our in vitro results and suggest that ADAM8 could serve as a potential regulator of synoviocyte invasion and migration activity in OA. Collectively, these results indicate that targeting ADAM8 in the synovium could potentially have a beneficial impact on the treatment of OA.

## Discussion

OA is a chronic disease characterized by degenerative changes in the knee cartilage, with or without bone hyperplasia and chronic synovitis [31, 32]. In a surveyed population in China, the prevalence of OA was high, reaching up to 8.1%. OA has become the leading cause of disability among middle-aged and elderly individuals in China [33, 34]. Unfortunately, this problem is only expected to worsen due to the aging population and increasing levels of obesity. This will inevitably lead to a significant burden on individuals, families, and society as a whole. The first line of treatment for OA in drug therapy is non-steroidal anti-inflammatory drugs. However, long-term use of these drugs can easily cause gastrointestinal reactions [35–37]. Preventing and effectively treating OA has become a significant challenge in the field of national public health management.

The synovium plays a crucial role in maintaining joint cavity homeostasis by communicating with the cartilage through an immune inflammatory network [38, 39]. In cases of OA, chronic and low-grade inflammation are the primary symptoms of synovial lesions [40]. Recent evidence suggests that synovial inflammation is a significant pathological feature in the early stages of OA and is closely linked to the patient’s clinical symptoms [41, 42]. When joints experience stress injuries, such as degeneration or trauma, the synovium is stimulated, leading to an inflammatory response. This results in the secretion of numerous inflammatory factors, such as IL-1, TNF-a, and IL-6, as well as matrix metalloproteinases (MMPs). These factors play a crucial role in the process of repairing articular cartilage injuries. The inflammatory microenvironment is a key pathological feature of tissue repair and is closely linked to tissue regeneration. IL-1β and other pro-inflammatory factors have the potential to disturb the equilibrium of chondrocytes, leading to their demise and gradual depletion of the cartilage matrix [43, 44]. Consequently, synovial inflammation is a crucial factor in the development of the pathological characteristics of OA.

FLSs are mesenchymal cells that reside in synovial tissue [45]. Recent studies have shown that the biological characteristics of FLSs in OA are similar to those in rheumatoid arthritis. This is due to changes in their behavior, which can lead to abnormal synovial hyperplasia [46]. Furthermore, FLSs have been identified as the primary inflammatory infiltrating cells in the synovium in OA [47]. Abnormally activated FLSs can accelerate the destruction of osteoarticular cartilage by releasing inflammatory factors, nitric oxide (NO), PGE2, and MMPs [48, 49]. Moreover, FLSs can activate receptors in the peripheral nervous system through inflammatory networks, which can worsen pain in OA patients [50]. This pain and cartilage damage can then escalate synovial inflammation, creating a vicious cycle that ultimately results in the loss of joint function.

In our experiment, we used IL-1β to interfere with FLSs to simulate the OA synovial inflammatory microenvironment. Transcriptome sequencing analysis revealed that the MAPK signaling pathway was activated during IL-1β-induced FLSs, accompanied by significant upregulation of ADAM8 gene. ADAM8 is a member of the ADAM family, which encodes disintegrin and metalloproteinase structural domains [51]. This protein has been implicated in various biological processes that involve interactions between cells and the extracellular matrix, as well as between cells themselves [52, 53]. These processes include fertilization, muscle development, and neurogenesis. Our study revealed that the expression levels of ADAM8 gradually increased with IL-1β stimulation in vitro. Additionally, we observed a significant increase in synovial ADAM8 expression in OA patients compared with ACL patients.

Previous studies have shown that ADAM8 plays a significant role in neutrophil migration and infiltration, which can help alleviate lung failure associated with acute respiratory distress syndrome [54]. ADAM8 has also been shown to play multiple roles in cancer cell migration, mechanics, and extracellular matrix remodeling. According to Liu et al., the activation of the NF-κB/MMP-13 signaling axis by ADAM8 resulted in the promotion of chondrosarcoma cell migration and invasion [26]. Koller also reported on ADAM8 as a possible emerging drug target treating of inflammatory and aggressive diseases [55]. In our study, we utilized a siRNA to suppress ADAM8 levels in FLS. Our findings indicate that ADAM8 inhibition resulted in a decrease in FLSs migration and invasion, which was accompanied by a significant reduction in MMPs. Additionally, we investigated the impact of ADAM8 inhibition on IL-1β-induced inflammation in FLSs. Our results demonstrate that ADAM8 blockade effectively reduced the expression of TNF-α, IL-6, COX2, and PGE2 in FLSs by regulating the MAPK signaling pathway. To further investigate the impact of ADAM8 knockdown on downstream signaling in IL-1β-stimulated FLSs, we conducted sequencing analysis. Our results revealed that cell migration signals were significantly downregulated, as evidenced by GO enrichment. Interestingly, FSCN1, a highly conserved actin binding protein, was among the downregulated migration-related differences that we identified. FSCN1 has been previously implicated in tumor cell growth and metastasis, further highlighting its potential role in regulating cell migration [56, 57]. Previous studies have established that FSCN1 plays a crucial role in regulating the biological function of FLSs [58]. In our research, we employed a potent inhibitor of FSCN1 in combination with siADAM8 as an intervention in IL-1β-stimulated FLSs. Our results indicate that the migration and migration ability of FLSs was further downregulated, indicating that ADAM8 may affect the biological function of FLSs by regulating FSCN1.

In our study, we conducted in vivo animal experiments in which we injected siADAM8 into the articular cavity of an OA rat model. Our findings showed that inhibiting ADAM8 effectively reduced OA progression and bone erosion in rats. Furthermore, our IHC staining revealed that inhibiting ADAM8 significantly reduced the expression level of IL-1β, IL-6, and TNF-α in the synovium of the rat knee. Previous studies conducted by Duan et al. have revealed that the notch1-ADAM8 pathway accelerates the progression of OA by promoting the degradation of chondrocytes through an extracellular mechanism [59]. In our study, we aimed to enhance the potential benefits of ADAM8 as a target in the treatment of OA by inhibiting its activity. Our findings shed new light on the treatment of OA by alleviating synovial inflammation.

Despite the valuable insights gained from our study, there are some limitations that should be acknowledged. One such limitation is that we did not include knockout mice, which could provide a more comprehensive understanding of the role of ADAM8 in regulating synovial biological phenotypes. Additionally, our experiment was conducted solely using the MIA-induced OA model, and further investigation is needed to determine the extent to which ADAM8 contributes to disease progression in older rats with OA. Our study indicates that the ADAM8/FSCN1/MAPK signaling axis may play a crucial role in the progression of OA-related synovitis. However, further investigation is required to verify other targets related to cell migration in the sequencing results and to fully understand the specific biological regulatory mechanism of ADAM8.

## Conclusions

Our study demonstrates the crucial role of MAPK in the inflammatory activation of FLSs, with the involvement of ADAM8. Targeted inhibition of ADAM8 expression inhibits the invasive and migratory ability of FLSs and also suppresses the release of inflammatory cytokines from FLSs. Our sequencing analysis suggests that there may be a potential involvement of FSCN1/MAPK signaling. In vitro, ADAM8 blockade was also observed to alleviate the progression of OA by effectively reducing synovial inflammation and MMP levels. Collectively, our findings indicate that ADAM8 administration could be a beneficial and viable therapeutic approach for treating OA.

### Supplementary Information

**Additional file 1: Table S1.** siRNA sequences of rat genes. **Table S2.** Primers used in qRT-PCR. **Figure S1.** qRT-PCR analysis of the mRNA expression levels of TULP2, DNAH6, CTRC, HIPK4, BEX1, CPA2, ARG2, BIN2A, MYCN and GNAL in FLSs after intervention with IL-1β. *P<0.05; **P<0.01. **Figure S2.** MAPK inhibitor Adezmapimod inhibited invasion, migration and inflammatory expression of IL-1β-stimulated FLSs. (A) CCK-8 results showing cytotoxicity of Adezmapimod on FLSs. (B) Western blot and quantitative analysis of P-JNK/JNK, P-ERK/ERK and P-P38/P38 in IL-1β-stimulated FLSs after treatment with Adezmapimod. (C, F) Wound healing assays and quantitative analysis of the migration ability of FLSs. (D, G) Transwell assays and quantitative analysis of the migration ability of FLSs. (E, H) Transwell assays and quantitative analysis of the invasion ability of FLSs with different concentrations of IL-1βin IL-1β-stimulated FLSs after treatment with Adezmapimod. (I) Western blot and quantitative analysis of IL-6, TNF-α and COX2 in IL-1β-stimulated FLSs after treatment with Adezmapimod. Scale bar = 200 µm. *P<0.05; **P<0.01. **Figure S3.** The GSEA analysis revealed that the MAPK signaling pathway was significantly enriched in FLSs after treatment with IL-1β. **Figure S4.** Western blot and quantitative analysis of P-JNK/JNK, P-ERK/ERK and P-P38/P38 in FLSs after treatment with BDP13176. ns, non-significant, **P<0.01.

## Data Availability

The datasets generated and/or analyzed during the current study are not publicly available but are available from the corresponding author on reasonable request.

## Abbreviations

OA: Osteoarthritis
FLS: Fibroblast-like synoviocytes
siRNA: Small interfering RNA
MMPs: Matrix metallomatrix proteinases
MAPK: Mitogen-activated protein kinase
ADAM8: A disintegrin and metalloproteinase domain-containing protein 8
FBS: Fetal bovine serum
PBS: Phosphate buffer saline
ACL: Anterior cruciate ligament injury
EDTA: Ethylenediaminetetraacetic acid
IHC: Immunohistochemical
H&E: Hematoxylin and eosin
DEGs: Differentially expressed genes
GO: Gene ontology
KEGG: Kyoto encyclopedia of genes and genomes

## References

[1] Xie Q, Yang K, Liang J, Shen Y, Liu L, Wang Y (2023). Spheroid culture of chondrocytes exhibits elevated levels of inflammation: a better approach for investigating the mechanisms of osteoarthritis?. Pharmacol Res.

[2] Abramoff B, Caldera FE (2020). Osteoarthritis: pathology, diagnosis, and treatment options. Med Clin North Am.

[3] Glyn-Jones S, Palmer AJ, Agricola R, Price AJ, Vincent TL, Weinans H, Carr AJ (2015). Osteoarthritis. Lancet.

[4] Martel-Pelletier J, Barr AJ, Cicuttini FM, Conaghan PG, Cooper C, Goldring MB, Goldring SR, Jones G, Teichtahl AJ, Pelletier JP (2016). Osteoarthritis. Nat Rev Dis Primers.

[5] Bijlsma JW, Berenbaum F, Lafeber FP (2011). Osteoarthritis: an update with relevance for clinical practice. Lancet.

[6] Kumar A, Palit P, Thomas S, Gupta G, Ghosh P, Goswami RP, Kumar Maity T, Dutta Choudhury M (2021). Osteoarthritis: prognosis and emerging therapeutic approach for disease management. Drug Dev Res.

[7] Yao Q, Wu X, Tao C, Gong W, Chen M, Qu M, Zhong Y, He T, Chen S, Xiao G (2023). Osteoarthritis: pathogenic signaling pathways and therapeutic targets. Signal Transduct Target Ther.

[8] Liu Y, Dzidotor G, Le TT, Vinikoor T, Morgan K, Curry EJ, Das R, McClinton A, Eisenberg E, Apuzzo LN (2022). Exercise-induced piezoelectric stimulation for cartilage regeneration in rabbits. Sci Transl Med.

[9] Berenbaum F, Wallace IJ, Lieberman DE, Felson DT (2018). Modern-day environmental factors in the pathogenesis of osteoarthritis. Nat Rev Rheumatol.

[10] Wu CL, Harasymowicz NS, Klimak MA, Collins KH, Guilak F (2020). The role of macrophages in osteoarthritis and cartilage repair. Osteoarthritis Cartilage.

[11] Mathiessen A, Conaghan PG (2017). Synovitis in osteoarthritis: current understanding with therapeutic implications. Arthritis Res Ther.

[12] Robinson WH, Lepus CM, Wang Q, Raghu H, Mao R, Lindstrom TM, Sokolove J (2016). Low-grade inflammation as a key mediator of the pathogenesis of osteoarthritis. Nat Rev Rheumatol.

[13] Griffin TM, Scanzello CR (2019). Innate inflammation and synovial macrophages in osteoarthritis pathophysiology. Clin Exp Rheumatol.

[14] Chen X, Gong W, Shao X, Shi T, Zhang L, Dong J, Shi Y, Shen S, Qin J, Jiang Q (2022). METTL3-mediated m(6)A modification of ATG7 regulates autophagy-GATA4 axis to promote cellular senescence and osteoarthritis progression. Ann Rheum Dis.

[15] Xiao Y, Ding L, Yin S, Huang Z, Zhang L, Mei W, Wu P, Wang P, Pan K (2021). Relationship between the pyroptosis of fibroblast-like synoviocytes and HMGB1 secretion in knee osteoarthritis. Mol Med Rep.

[16] Bustamante MF, Oliveira PG, Garcia-Carbonell R, Croft AP, Smith JM, Serrano RL, Sanchez-Lopez E, Liu X, Kisseleva T, Hay N (2018). Hexokinase 2 as a novel selective metabolic target for rheumatoid arthritis. Ann Rheum Dis.

[17] Garcia-Carbonell R, Divakaruni AS, Lodi A, Vicente-Suarez I, Saha A, Cheroutre H, Boss GR, Tiziani S, Murphy AN, Guma M (2016). Critical role of glucose metabolism in rheumatoid arthritis fibroblast-like synoviocytes. Arthritis Rheumatol.

[18] Xu L, Peng Q, Xuan W, Feng X, Kong X, Zhang M, Tan W, Xue M, Wang F (2016). Interleukin-29 enhances synovial inflammation and cartilage degradation in osteoarthritis. Mediators Inflamm.

[19] Min HK, Won JY, Kim BM, Lee KA, Lee SJ, Lee SH, Kim HR, Kim KW (2020). Interleukin (IL)-25 suppresses IL-22-induced osteoclastogenesis in rheumatoid arthritis via STAT3 and p38 MAPK/IκBα pathway. Arthritis Res Ther.

[20] Sluzalska KD, Liebisch G, Lochnit G, Ishaque B, Hackstein H, Schmitz G, Rickert M, Steinmeyer J (2017). Interleukin-1β affects the phospholipid biosynthesis of fibroblast-like synoviocytes from human osteoarthritic knee joints. Osteoarthritis Cartilage.

[21] Conrad C, Yildiz D, Cleary SJ, Margraf A, Cook L, Schlomann U, Panaretou B, Bowser JL, Karmouty-Quintana H, Li J (2022). ADAM8 signaling drives neutrophil migration and ARDS severity. JCI Insight.

[22] Conrad C, Benzel J, Dorzweiler K, Cook L, Schlomann U, Zarbock A, Slater EP, Nimsky C, Bartsch JW (2019). ADAM8 in invasive cancers: links to tumor progression, metastasis, and chemoresistance. Clin Sci (Lond).

[23] Awan T, Babendreyer A, Mahmood Alvi A, Düsterhöft S, Lambertz D, Bartsch JW, Liedtke C, Ludwig A (2021). Expression levels of the metalloproteinase ADAM8 critically regulate proliferation, migration and malignant signalling events in hepatoma cells. J Cell Mol Med.

[24] Knolle MD, Owen CA (2009). ADAM8: a new therapeutic target for asthma. Expert Opin Ther Targets.

[25] Awan T, Babendreyer A, Wozniak J, Alvi AM, Sterzer V, Cook L, Bartsch JW, Liedtke C, Yildiz D, Ludwig A (2021). Expression of the metalloproteinase ADAM8 is upregulated in liver inflammation models and enhances cytokine release in vitro. Mediators Inflamm.

[26] Liu Y, Li ZH, Zhang L, Lu SB (2019). ADAM8 promotes chondrosarcoma cell migration and invasion by activating the NF-κB/MMP-13 signaling axis. Anticancer Drugs.

[27] Barker T, Rogers VE, Henriksen VT, Trawick RH, Momberger NG, Lynn Rasmussen G (2021). Circulating IL-10 is compromised in patients predisposed to developing and in patients with severe knee osteoarthritis. Sci Rep.

[28] Alonso B, Bravo B, Mediavilla L, Gortazar AR, Forriol F, Vaquero J, Guisasola MC (2020). Osteoarthritis-related biomarkers profile in chronic anterior cruciate ligament injured knee. Knee.

[29] Machner A, Baier A, Wille A, Drynda S, Pap G, Drynda A, Mawrin C, Bühling F, Gay S, Neumann W (2003). Higher susceptibility to Fas ligand induced apoptosis and altered modulation of cell death by tumor necrosis factor-alpha in periarticular tenocytes from patients with knee joint osteoarthritis. Arthritis Res Ther.

[30] Hu Y, Gui Z, Zhou Y, Xia L, Lin K, Xu Y (2019). Quercetin alleviates rat osteoarthritis by inhibiting inflammation and apoptosis of chondrocytes, modulating synovial macrophages polarization to M2 macrophages. Free Radic Biol Med.

[31] Cai W, Li H, Zhang Y, Han G (2020). Identification of key biomarkers and immune infiltration in the synovial tissue of osteoarthritis by bioinformatics analysis. PeerJ.

[32] Kalaitzoglou E, Griffin TM, Humphrey MB (2017). Innate immune responses and osteoarthritis. Curr Rheumatol Rep.

[33] Marks R, Allegrante JP (2005). Chronic osteoarthritis and adherence to exercise: a review of the literature. J Aging Phys Act.

[34] Buckwalter JA, Saltzman C, Brown T (2004). The impact of osteoarthritis: implications for research. Clin Orthop Relat Res.

[35] Bartels EM, Juhl CB, Christensen R, Hagen KB, Danneskiold-Samsøe B, Dagfinrud H, Lund H (2016). Aquatic exercise for the treatment of knee and hip osteoarthritis. Cochrane Database Syst Rev.

[36] Adatia A, Rainsford KD, Kean WF (2012). Osteoarthritis of the knee and hip. Part II: therapy with ibuprofen and a review of clinical trials. J Pharm Pharmacol.

[37] Pavelka K, Bruyère O, Cooper C, Kanis JA, Leeb BF, Maheu E, Martel-Pelletier J, Monfort J, Pelletier JP, Rizzoli R (2016). Diacerein: benefits, risks and place in the management of osteoarthritis. An opinion-based report from the ESCEO. Drugs Aging.

[38] Sanchez-Lopez E, Coras R, Torres A, Lane NE, Guma M (2022). Synovial inflammation in osteoarthritis progression. Nat Rev Rheumatol.

[39] Mapp PI, Walsh DA (2012). Mechanisms and targets of angiogenesis and nerve growth in osteoarthritis. Nat Rev Rheumatol.

[40] Liu-Bryan R (2013). Synovium and the innate inflammatory network in osteoarthritis progression. Curr Rheumatol Rep.

[41] Scanzello CR, Goldring SR (2012). The role of synovitis in osteoarthritis pathogenesis. Bone.

[42] Roebuck MM, Jamal J, Lane B, Wood A, Santini A, Wong PF, Bou-Gharios G, Frostick SP (2022). Cartilage debris and osteoarthritis risk factors influence gene expression in the synovium in end stage osteoarthritis. Knee.

[43] Wang T, He C (2018). Pro-inflammatory cytokines: The link between obesity and osteoarthritis. Cytokine Growth Factor Rev.

[44] Adams SB, Setton LA, Kensicki E, Bolognesi MP, Toth AP, Nettles DL (2012). Global metabolic profiling of human osteoarthritic synovium. Osteoarthritis Cartilage.

[45] Hu T, Zhang Z, Deng C, Ma X, Liu X (2022). Effects of β2 integrins on osteoclasts, macrophages, chondrocytes, and synovial fibroblasts in osteoarthritis. Biomolecules.

[46] Wu Y, Li Z, Jia W, Li M, Tang M (2019). Upregulation of stanniocalcin-1 inhibits the development of osteoarthritis by inhibiting survival and inflammation of fibroblast-like synovial cells. J Cell Biochem.

[47] Knights AJ, Farrell EC, Ellis OM, Lammlin L, Junginger LM, Rzeczycki PM, Bergman RF, Pervez R, Cruz M, Knight E (2023). Synovial fibroblasts assume distinct functional identities and secrete R-spondin 2 in osteoarthritis. Ann Rheum Dis.

[48] Tetlow LC, Woolley DE (2004). Effect of histamine on the production of matrix metalloproteinases-1, -3, -8 and -13, and TNFalpha and PGE(2) by human articular chondrocytes and synovial fibroblasts in vitro: a comparative study. Virchows Arch.

[49] Lee YA, Choi HM, Lee SH, Yang HI, Yoo MC, Hong SJ, Kim KS (2012). Synergy between adiponectin and interleukin-1β on the expression of interleukin-6, interleukin-8, and cyclooxygenase-2 in fibroblast-like synoviocytes. Exp Mol Med.

[50] Hammaker D, Firestein GS (2018). Epigenetics of inflammatory arthritis. Curr Opin Rheumatol.

[51] Le HT, Atif J, Mara DL, Castellana B, Treissman J, Baltayeva J, Beristain AG (2018). ADAM8 localizes to extravillous trophoblasts within the maternal-fetal interface and potentiates trophoblast cell line migration through a β1 integrin-mediated mechanism. Mol Hum Reprod.

[52] Mierke CT (2023). The versatile roles of ADAM8 in cancer cell migration, mechanics, and extracellular matrix remodeling. Front Cell Dev Biol.

[53] Knolle MD, Nakajima T, Hergrueter A, Gupta K, Polverino F, Craig VJ, Fyfe SE, Zahid M, Permaul P, Cernadas M (2013). Adam8 limits the development of allergic airway inflammation in mice. J Immunol.

[54] Yu X, Shi J, Wang X, Zhang F (2020). Propofol affects the growth and metastasis of pancreatic cancer via ADAM8. Pharmacol Rep.

[55] Koller G, Schlomann U, Golfi P, Ferdous T, Naus S, Bartsch JW (2009). ADAM8/MS2/CD156, an emerging drug target in the treatment of inflammatory and invasive pathologies. Curr Pharm Des.

[56] Liu C, Gao H, Cao L, Gui S, Liu Q, Li C, Li D, Gong L, Zhang Y (2016). The role of FSCN1 in migration and invasion of pituitary adenomas. Mol Cell Endocrinol.

[57] Xiao P, Liu W, Zhou H (2021). [Retracted] miR-200b inhibits migration and invasion in non-small cell lung cancer cells via targeting FSCN1. Mol Med Rep.

[58] Chen SY, Hsieh JL, Wu PT, Shiau AL, Wu CL (2022). MicroRNA-133 suppresses cell viability and migration of rheumatoid arthritis fibroblast-like synoviocytes by down-regulation of MET, EGFR, and FSCN1 expression. Mol Cell Biochem.

[59] Duan B, Liu Y, Hu H, Shi FG, Liu YL, Xue H, Yun XY, Yan MY, Han XR, Chen AF (2019). Notch1-ADAM8 positive feed-back loop regulates the degradation of chondrogenic extracellular matrix and osteoarthritis progression. Cell Commun Signal.

