# Supplementary material for: ADAM8 silencing suppresses the migration and invasion of fibroblast-like synoviocytes via FSCN1/MAPK cascade in osteoarthritis
Source: Arthritis Res Ther. 2024 Jan 13;26:20. doi: 10.1186/s13075-023-03238-w (PMC10787439; doi:10.1186/s13075-023-03238-w)
Supplement: Supplementary file 1 — Additional file 1: Table S1. siRNA sequences of rat genes. Table S2. Primers used in qRT-PCR. Figure S1. qRT-PCR analysis of the mRNA expression levels of TULP2, DNAH6, CTRC, HIPK4, BEX1, CPA2, ARG2, BIN2A, MYCN and GNAL in FLSs after intervention with IL-1β. *P<0.05; **P<0.01. Figure S2. MAPK inhibitor Adezmapimod inhibited invasion, migration and inflammatory expression of IL-1β-stimulated FLSs. (A) CCK-8 results showing cytotoxicity of Adezmapimod on FLSs. (B) Western blot and quantitative analysis of P-JNK/JNK, P-ERK/ERK and P-P38/P38 in IL-1β-stimulated FLSs after treatment with Adezmapimod. (C, F) Wound healing assays and quantitative analysis of the migration ability of FLSs. (D, G) Transwell assays and quantitative analysis of the migration ability of FLSs. (E, H) Transwell assays and quantitative analysis of the invasion ability of FLSs with different concentrations of IL-1βin IL-1β-stimulated FLSs after treatment with Adezmapimod. (I) Western blot and quantitative analysis of IL-6, TNF-α and COX2 in IL-1β-stimulated FLSs after treatment with Adezmapimod. Scale bar = 200 µm. *P<0.05; **P<0.01. Figure S3. The GSEA analysis revealed that the MAPK signaling pathway was significantly enriched in FLSs after treatment with IL-1β. Figure S4. Western blot and quantitative analysis of P-JNK/JNK, P-ERK/ERK and P-P38/P38 in FLSs after treatment with BDP13176. ns, non-significant, **P<0.01. [file 13075_2023_3238_MOESM1_ESM.zip › Supplementary Material 0812.docx]

**Title Page**

**Title of the article**

ADAM8 silencing suppresses the migration and invasion of fibroblast-like synoviocytes via FSCN1/MAPK cascade in osteoarthritis

**Running Title:** ADAM8 regulate OA-FLS via FSCN1/MAPK pathway

**The name(s) of the author(s)**

Kai Chen^1,3,a^, MS; Huaqiang Tao^1,a^, MS; Pengfei Zhu^1,a^, MS; Miao Chu^1,a^, MS; Xueyan Li^2^, MS; Yi Shi^2^, MS; Liyuan Zhang^2^, MS; Yaozeng Xu^1^, PhD; Shujun Lv^3,*^, PhD; Lixin Huang^1,*^, PhD; Huang Wei^4,*^; Dechun Geng^1,*^, PhD.

**Name and address of the institution**

^1^ Department of Orthopedics, The First Affiliated Hospital of Soochow University, No. 188 Shizi Street, Suzhou, Jiangsu, China.

^2^ Anesthesiology department, Suzhou Municipal Hospital (North District), Nanjing Medical University Affiliated Suzhou Hospital, 242, Guangjj Road, Suzhou, Jiangsu, China.

^3^ Department of Orthopedics, Hai’an People's Hospital, Zhongba Road 17，Hai’an, Jiangsu, China.

^4^ Department of Orthopaedics, The First Affiliated Hospital of USTC, Division of Life Sciences and Medicine, University of Science and Technology of China, Lujiang Road 17, Hefei, An’hui, China.

^a^ **Contribute equally to this work**

* **Correspondence and requests for materials should be addressed to**

Lixin Huang Email: szhuanglx@yeah.net;

Huang Wei Email: zgkdhwei@ustc.edu.cn;

Dechun Geng Email: [szgengdc@suda.edu.cn](mailto:szgengdc@suda.edu.cn).

Supplementary Material

Table S1. siRNA sequences of rat genes

| Gene | Sense | Antisense |
| --- | --- | --- |
| siADAM8 1 | 5’-GCCAAUCCGGAAUCUGCAUTT-3’ | 5’-AUGCAGAUUCCGGAUUGGCTT-3’ |
| siADAM8 2 | 5’-GCGUGCUGUCUCGGAACAATT-3’ | 5’-UUGUUCCGAGACAGCACGCTT-3’ |
| siADAM8 3  siNC | 5’-GCCUACCUGCUCAUGGAAUTT-3’  5’-UUCUCCGAACGUGUCACGUTT-3’ | 5’-AUUCCAUGAGCAGGUAGGCTT-3’  5’-ACGUGACACGUUCGGAGAATT-3’ |

Table S2. Primers used in qRT-PCR.

| Gene | Primer sequence (F) | Primer sequence (R) |
| --- | --- | --- |
| MMP-1 | CTTCCAATACGTGCAGCAGA | TCTTCAGGGCTTTCTCGTTC |
| MMP-13  ADAM8  TNF-α  IL-6  PGE2  COX2  GNAL  MYCN  BIN2A  ARG2  CPA2  BEX1  HIPK4  CTRC  DNAH6  TULP2 | GGAAATCATCCCTTGCACGC  GGGCTGCTATGGGAGTATGTACC  TGCCCCTCATCAGGCCCAGT  TCCAGACTCCGATCATCAAGC  GCTGTACCAGTGCAGGACCTCAT  GCTGTACCAGTGCAGGACCTCAT  AAGCAGATCCACGTCAGCAACAG  ACCCTGCCTACCGACCTTTCC  TGAGGAGTTGAAAGGTGGCGTAAAG  CGGCTGATTGGCAAGAGGAAGAG  GCAAAGAGAGCGTGGTGGGAAC  AGGGCAGGATGAGAGAGGAGAATG  CAACCAAGCCAGTCGTTCAGGAG  GAATTACGGTCCTCGCTGCCATC  AGCCTTGAAACCCACCCGAAATG  CTGCTGCTATGCCTAACACACCTC | TGTTTGAACTTGGTGGGGCT  TAGTGTTGCTGTCAGTCTGGAGAG  GCTCGGCTGGGACTCGTGTT  GCTCATGGTGTTCAGAATTGTGT  CTCTCCTGAGAGTGAGATCACACGA  GCTGTACCAGTGCAGGACCTCAT  CACCAAGGCACGGAACTCTACAAG  GAGGAGGAGCGTCTCTTCTCTACC  CAAGTGAGAAGGTGGATGGTGGAAC  GGAAGGTGGTCATAGGCAATGTGTC  GATTGGTGACAGGCAGCAGGAAG  TAGAAGCTGGCAGAACTGGAGACC  CCAGGTAGAACTTGAGGGCATCATG  AGCCTTCCTCATCCTCCACTGTC  CCGCACCGCAGTTATGGATGG  GGTCACTTCCTCCACATCTGAATCG |
| GAPDH | GGTTGTCTCCTGCGACTTCA | TGGTCCAGGGTTTCTTACTCC |


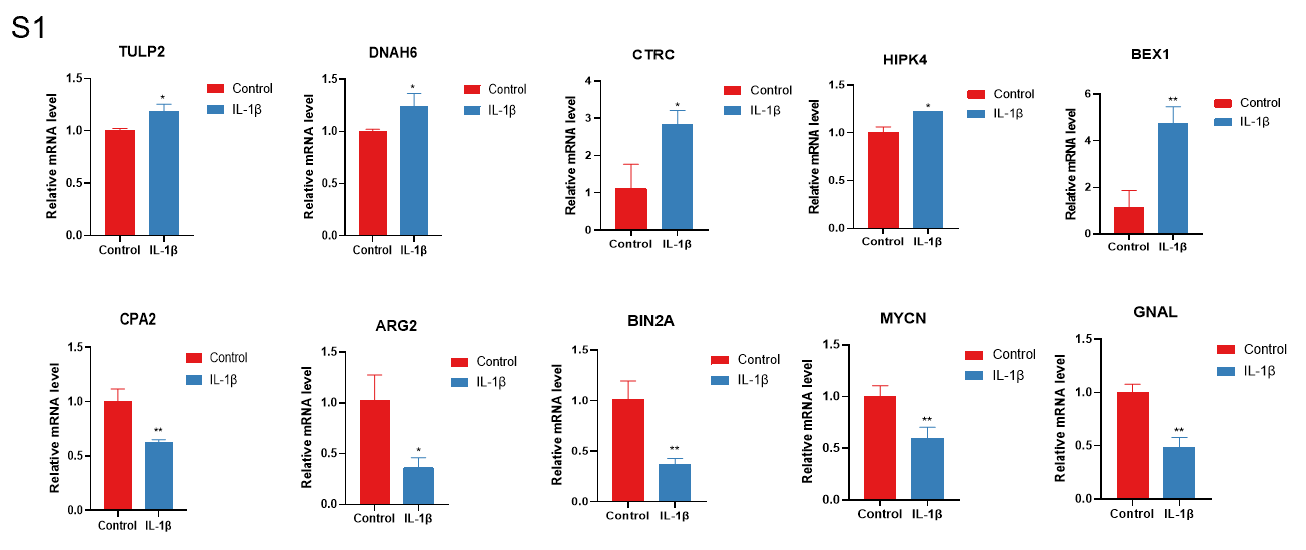


Figure S1. qRT-PCR analysis of the mRNA expression levels of TULP2, DNAH6, CTRC, HIPK4, BEX1, CPA2, ARG2, BIN2A, MYCN and GNAL in FLSs after intervention with IL-1β. *P<0.05; **P<0.01.

**
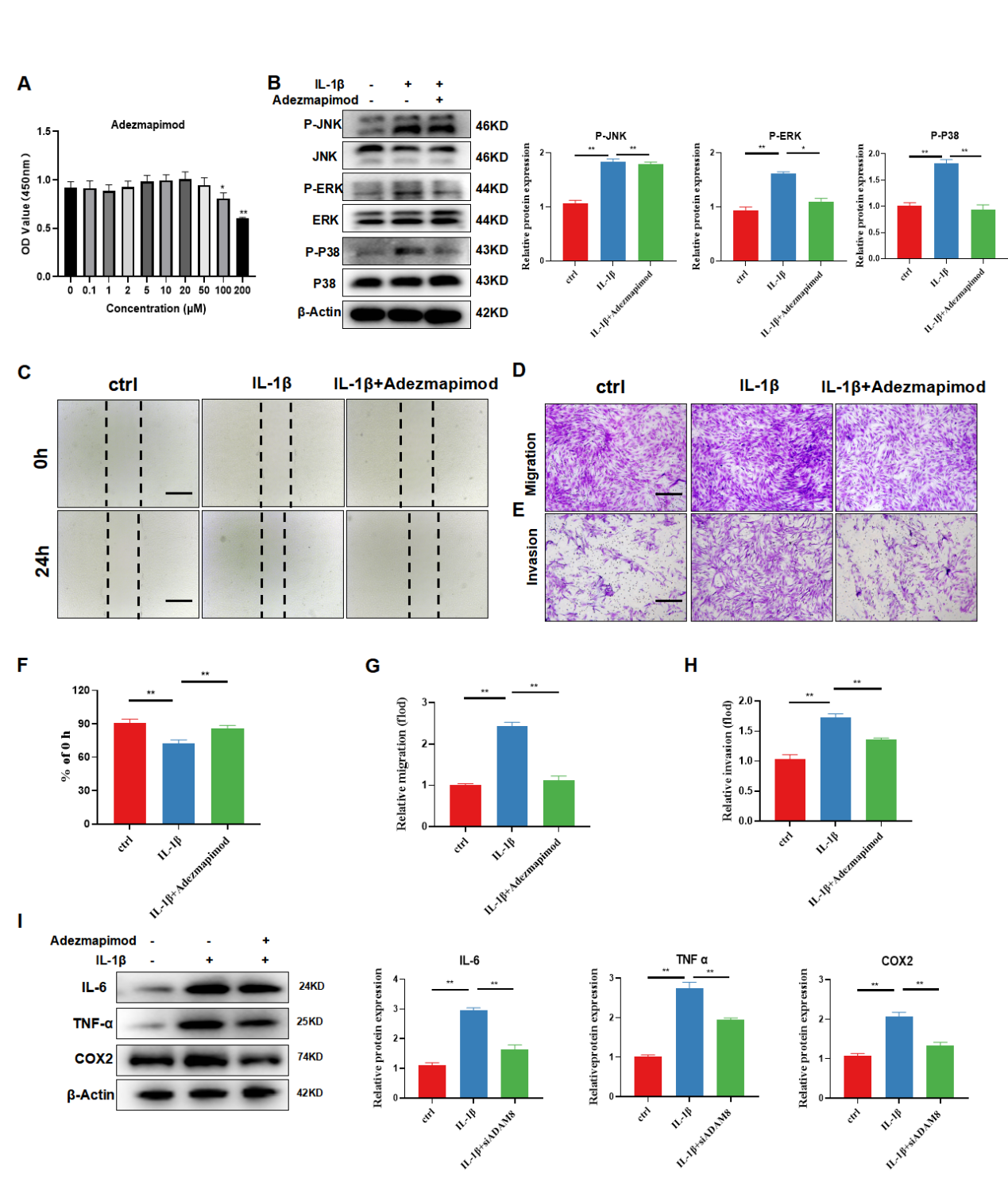
**

Figure S2. MAPK inhibitor Adezmapimod inhibited invasion, migration and inflammatory expression of IL-1β-stimulated FLSs. (A) CCK-8 results showing cytotoxicity of Adezmapimod on FLSs. (B) Western blot and quantitative analysis of P-JNK/JNK, P-ERK/ERK and P-P38/P38 in IL-1β-stimulated FLSs after treatment with Adezmapimod. (C, F) Wound healing assays and quantitative analysis of the migration ability of FLSs. (D, G) Transwell assays and quantitative analysis of the migration ability of FLSs. (E, H) Transwell assays and quantitative analysis of the invasion ability of FLSs with different concentrations of IL-1βin IL-1β-stimulated FLSs after treatment with Adezmapimod. (I) Western blot and quantitative analysis of IL-6, TNF-α and COX2 in IL-1β-stimulated FLSs after treatment with Adezmapimod. Scale bar = 200 µm. *P<0.05; **P<0.01.


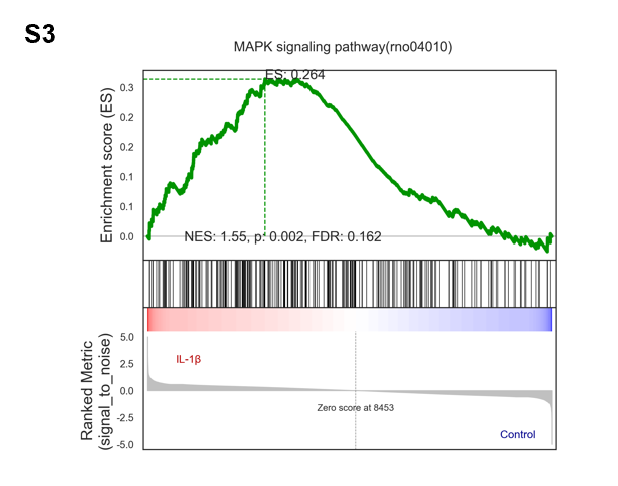


Figure S3. The GSEA analysis revealed that the MAPK signaling pathway was significantly enriched in FLSs after treatment with IL-1β.


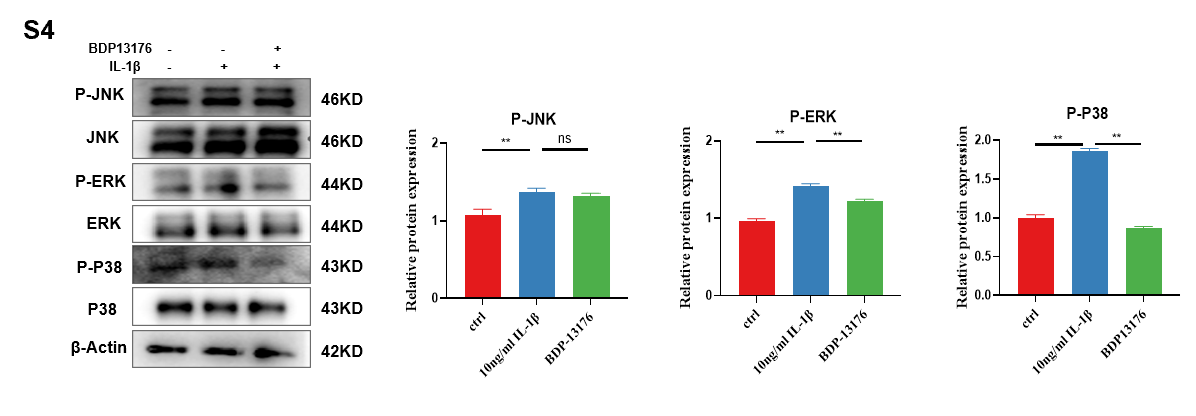


Figure S4. Western blot and quantitative analysis of P-JNK/JNK, P-ERK/ERK and P-P38/P38 in FLSs after treatment with BDP13176. ns, non-significant, **P<0.01.
