# Supplementary figures and images for: ADAM8 silencing suppresses the migration and invasion of fibroblast-like synoviocytes via FSCN1/MAPK cascade in osteoarthritis
Source: Arthritis Res Ther. 2024 Jan 13;26:20. doi: 10.1186/s13075-023-03238-w (PMC10787439; doi:10.1186/s13075-023-03238-w)

MMP1

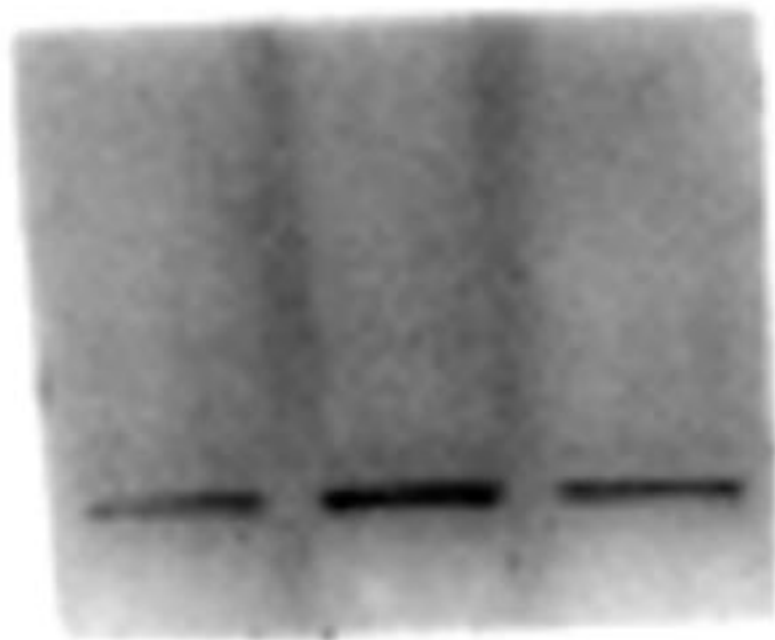

ACTIN

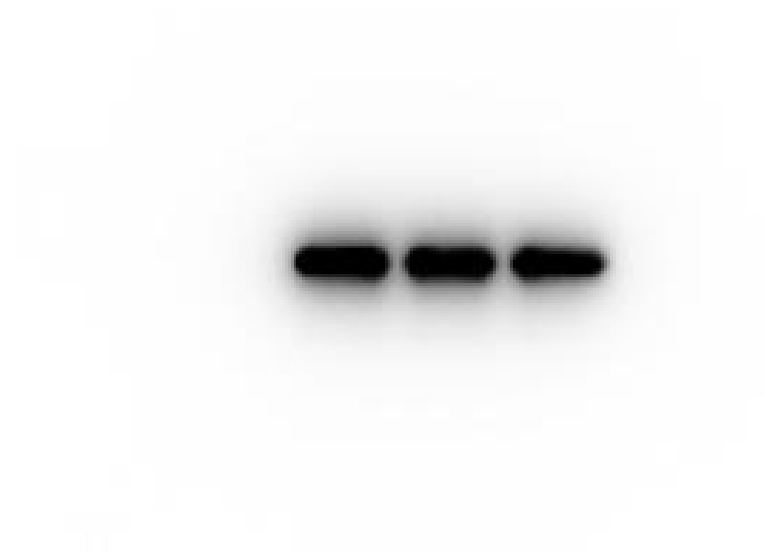

MMP13

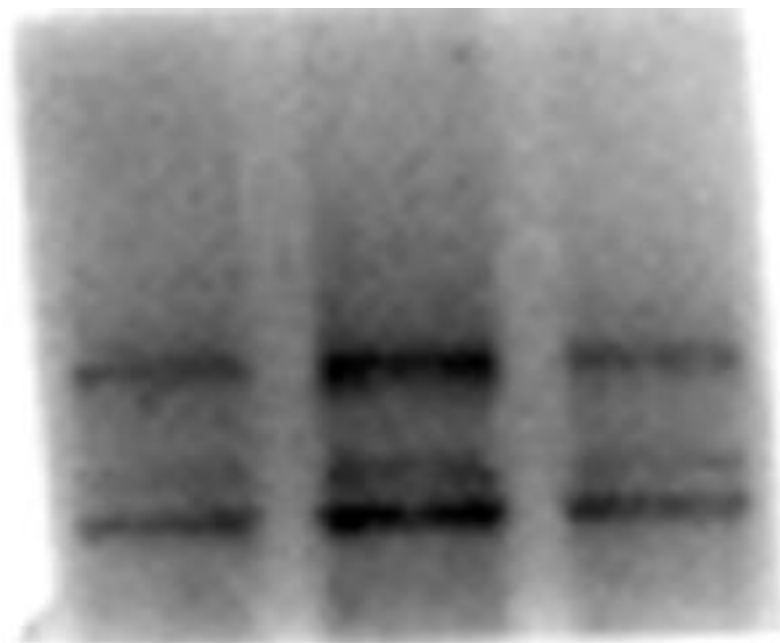

COX2

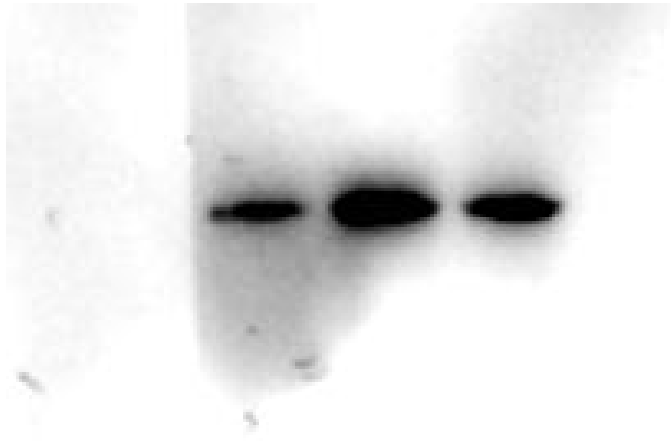

ACTIN

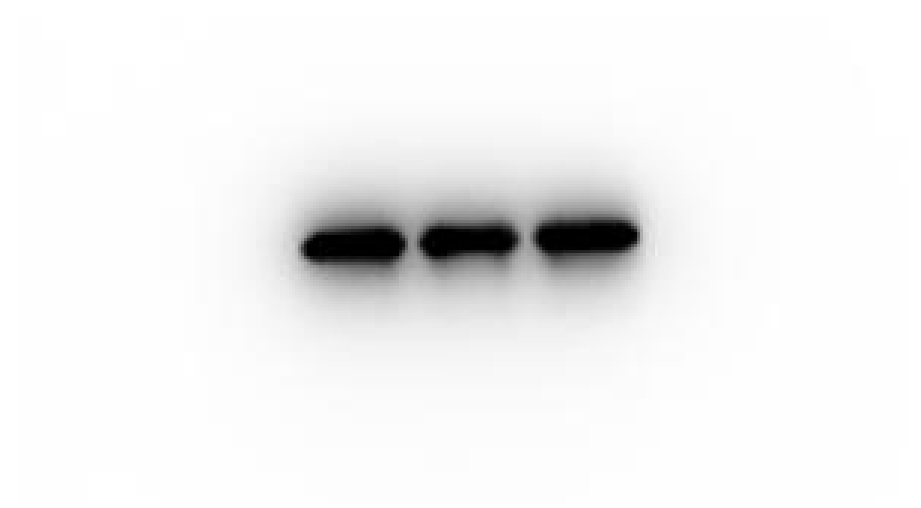

TNFA

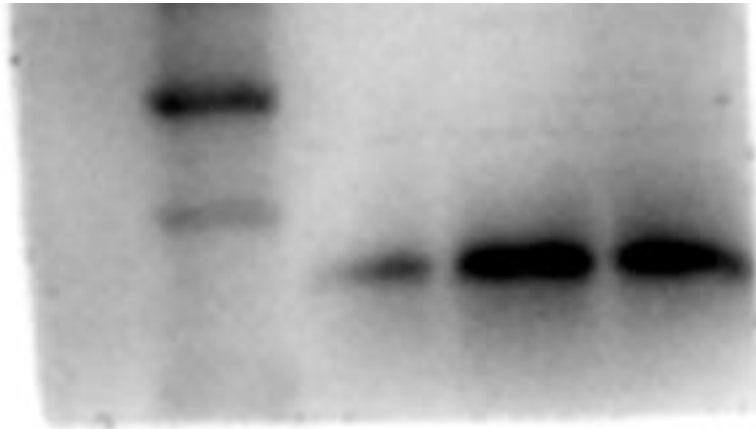

IL6

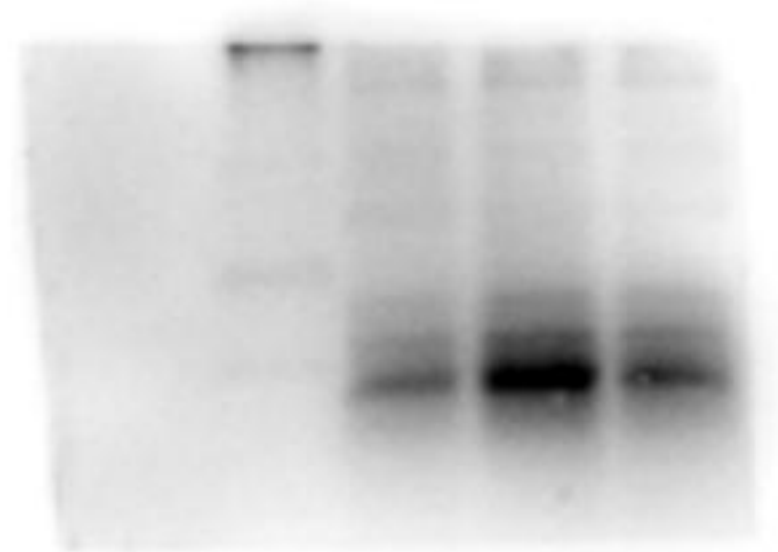

ERK

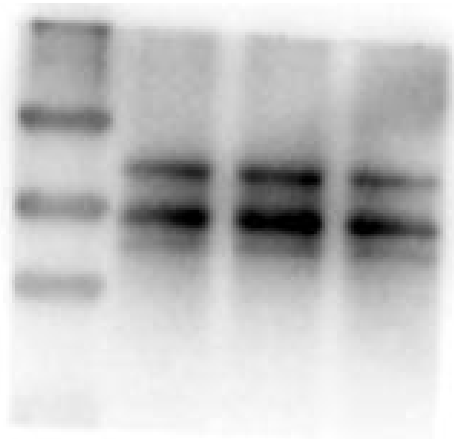

P38

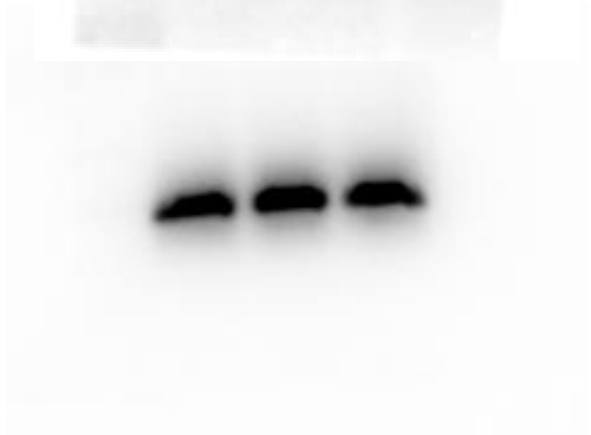

JNK

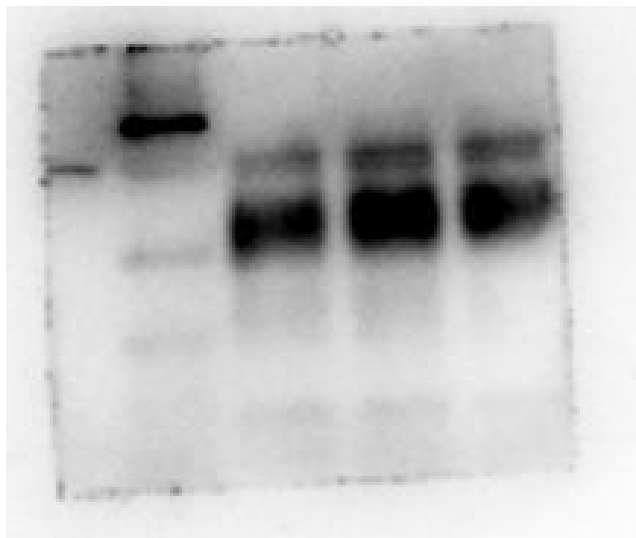

P-ERK

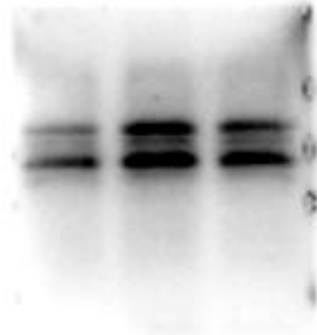

P-P38

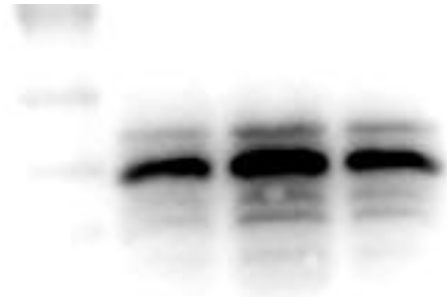

P-JNK

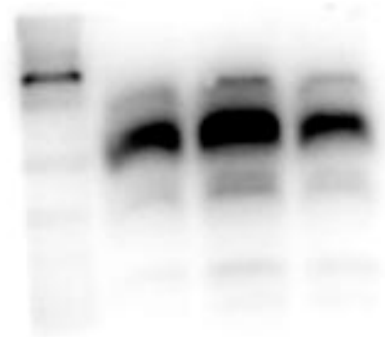

ACTIN

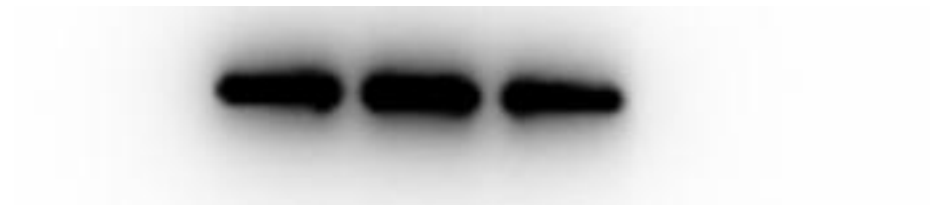

Supplement: Supplementary file 1 — Additional file 1: Table S1. siRNA sequences of rat genes. Table S2. Primers used in qRT-PCR. Figure S1. qRT-PCR analysis of the mRNA expression levels of TULP2, DNAH6, CTRC, HIPK4, BEX1, CPA2, ARG2, BIN2A, MYCN and GNAL in FLSs after intervention with IL-1β. *P<0.05; **P<0.01. Figure S2. MAPK inhibitor Adezmapimod inhibited invasion, migration and inflammatory expression of IL-1β-stimulated FLSs. (A) CCK-8 results showing cytotoxicity of Adezmapimod on FLSs. (B) Western blot and quantitative analysis of P-JNK/JNK, P-ERK/ERK and P-P38/P38 in IL-1β-stimulated FLSs after treatment with Adezmapimod. (C, F) Wound healing assays and quantitative analysis of the migration ability of FLSs. (D, G) Transwell assays and quantitative analysis of the migration ability of FLSs. (E, H) Transwell assays and quantitative analysis of the invasion ability of FLSs with different concentrations of IL-1βin IL-1β-stimulated FLSs after treatment with Adezmapimod. (I) Western blot and quantitative analysis of IL-6, TNF-α and COX2 in IL-1β-stimulated FLSs after treatment with Adezmapimod. Scale bar = 200 µm. *P<0.05; **P<0.01. Figure S3. The GSEA analysis revealed that the MAPK signaling pathway was significantly enriched in FLSs after treatment with IL-1β. Figure S4. Western blot and quantitative analysis of P-JNK/JNK, P-ERK/ERK and P-P38/P38 in FLSs after treatment with BDP13176. ns, non-significant, **P<0.01. [file 13075_2023_3238_MOESM1_ESM.zip › original western blot.pdf]

IL-6

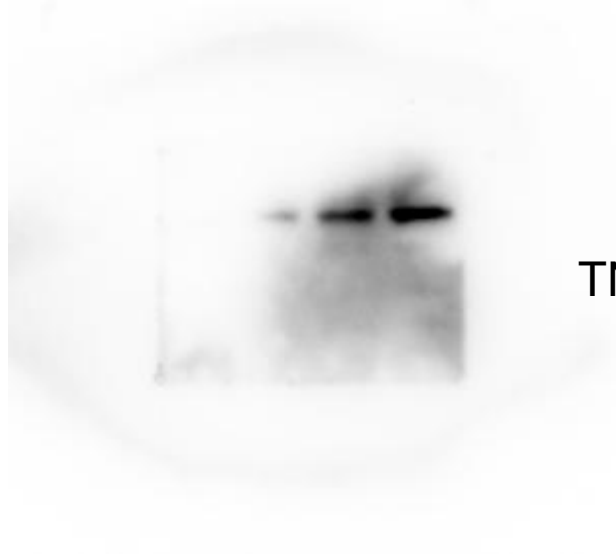

TNFA

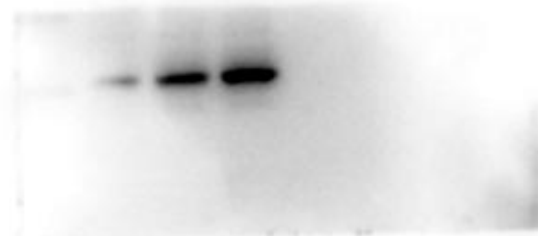

COX2

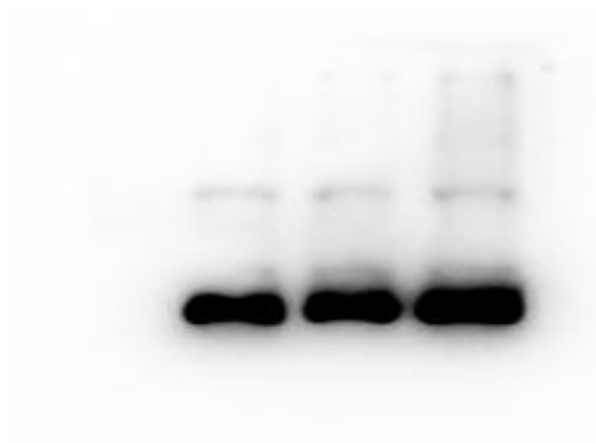

ACTIN

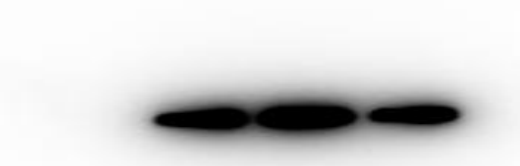

ACTIN

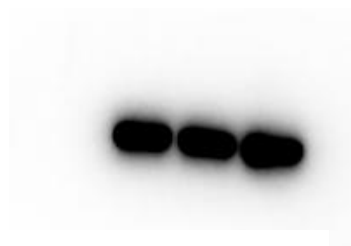

P38

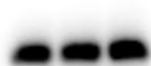

P-  
P38

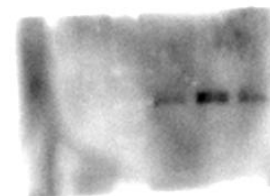

ERK

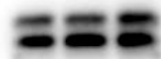

P-  
ERK

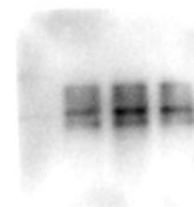

JNK

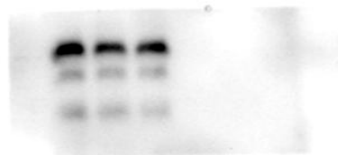

P-  
JNK

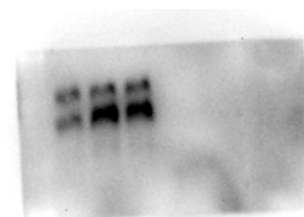

actin

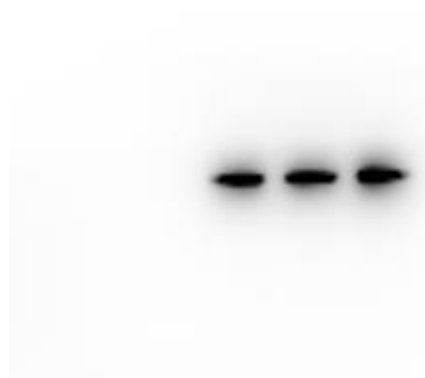

TNF  
A

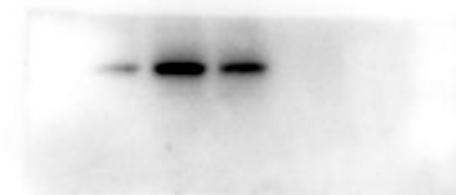

IL-6

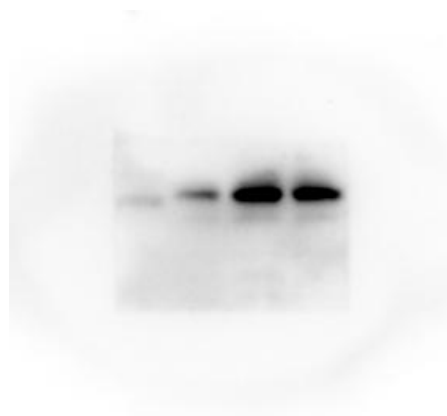

COX  
2

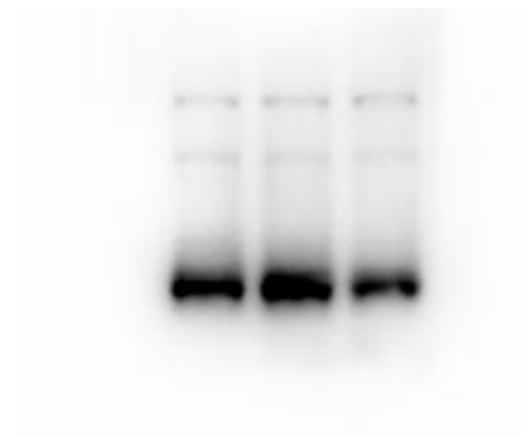

ACTIN

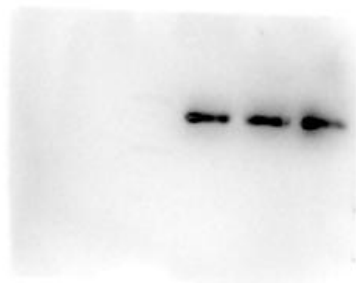

P38

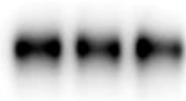

P-  
P38

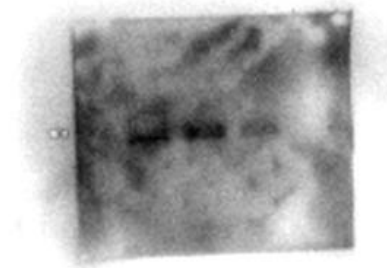

ERK

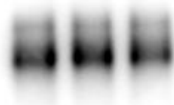

P-  
ERK

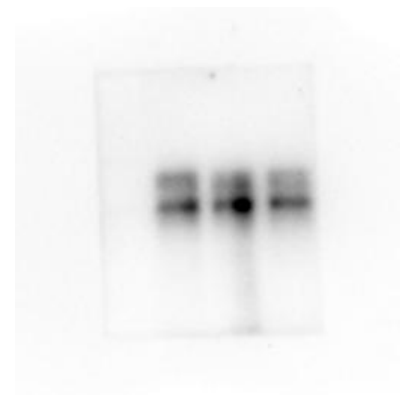

JNK

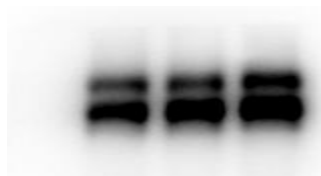

P-  
JNK

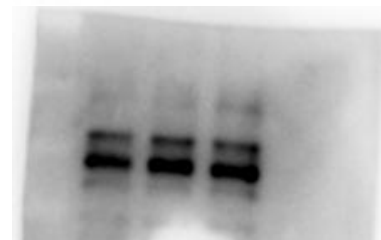

Supplement: Supplementary file 1 — Additional file 1: Table S1. siRNA sequences of rat genes. Table S2. Primers used in qRT-PCR. Figure S1. qRT-PCR analysis of the mRNA expression levels of TULP2, DNAH6, CTRC, HIPK4, BEX1, CPA2, ARG2, BIN2A, MYCN and GNAL in FLSs after intervention with IL-1β. *P<0.05; **P<0.01. Figure S2. MAPK inhibitor Adezmapimod inhibited invasion, migration and inflammatory expression of IL-1β-stimulated FLSs. (A) CCK-8 results showing cytotoxicity of Adezmapimod on FLSs. (B) Western blot and quantitative analysis of P-JNK/JNK, P-ERK/ERK and P-P38/P38 in IL-1β-stimulated FLSs after treatment with Adezmapimod. (C, F) Wound healing assays and quantitative analysis of the migration ability of FLSs. (D, G) Transwell assays and quantitative analysis of the migration ability of FLSs. (E, H) Transwell assays and quantitative analysis of the invasion ability of FLSs with different concentrations of IL-1βin IL-1β-stimulated FLSs after treatment with Adezmapimod. (I) Western blot and quantitative analysis of IL-6, TNF-α and COX2 in IL-1β-stimulated FLSs after treatment with Adezmapimod. Scale bar = 200 µm. *P<0.05; **P<0.01. Figure S3. The GSEA analysis revealed that the MAPK signaling pathway was significantly enriched in FLSs after treatment with IL-1β. Figure S4. Western blot and quantitative analysis of P-JNK/JNK, P-ERK/ERK and P-P38/P38 in FLSs after treatment with BDP13176. ns, non-significant, **P<0.01. [file 13075_2023_3238_MOESM1_ESM.zip › revision original western blot.pdf]
